# Supplementary material for: Chemical Constituents from Ficus sagittifolia Stem Bark and Their Antimicrobial Activities
Source: Plants (Basel). 2023 Jul 28;12(15):2801. doi: 10.3390/plants12152801 (PMC10420693; doi:10.3390/plants12152801)
Supplement: Supplementary file 1 [file plants-12-02801-s001.zip › plants-2498129-supplementary.pdf]

Supplementary Material

Chemical constituents from *Ficus sagittifolia* stem bark and their antimicrobial activities

Olayombo M. Taiwo [1, 2*], Olaoluwa O. Olaoluwa [1], Olapeju O. Aiyelaagbe [1] and Thomas J. Schmidt [2,*]

[1]Department of Chemistry, University of Ibadan, Ibadan, 200284, Nigeria; tolayombo@gmail.com (O.M.T.); omosalewa.olaoluwa@gmail.com (O.O.O.); oaiyelaagbe@gmail.com (O.O.A.)

[2]Institute of Pharmaceutical Biology and Phytochemistry (IPBP), University of Muenster, PharmaCampus, Correnstrasse 48, D-48149, Germany; thomschm@uni-muenster.de (T.J.S.); tolayombo@gmail.com (O.M.T.)

*Correspondence: thomschm@uni-muenster.de (T.J.S.); tolayombo@gmail.com (O.M.T.)

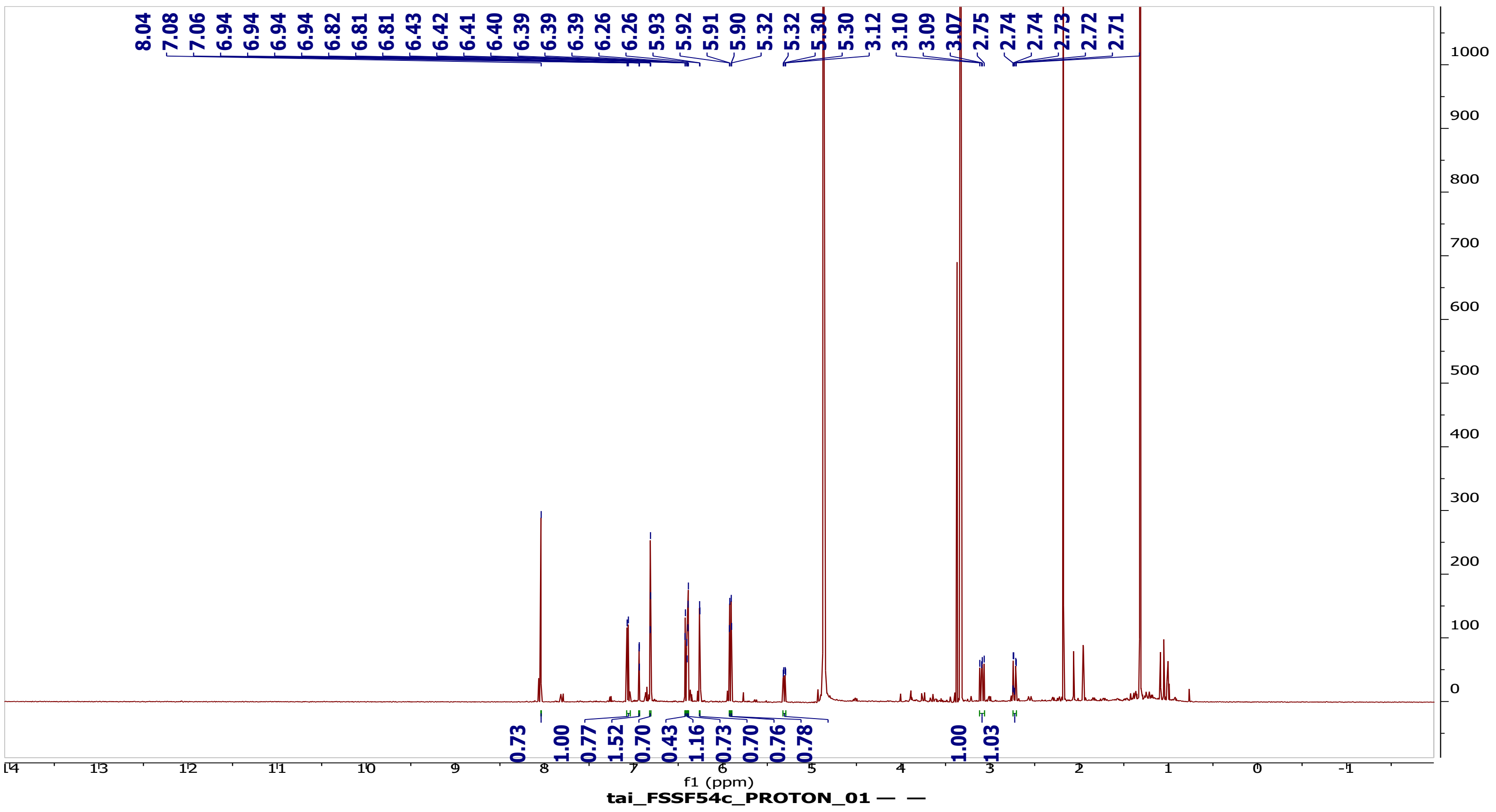

Figure S-1. ^{1}H-NMR spectrum of compounds **1** and **2** (600 MHz, CD_3OD)

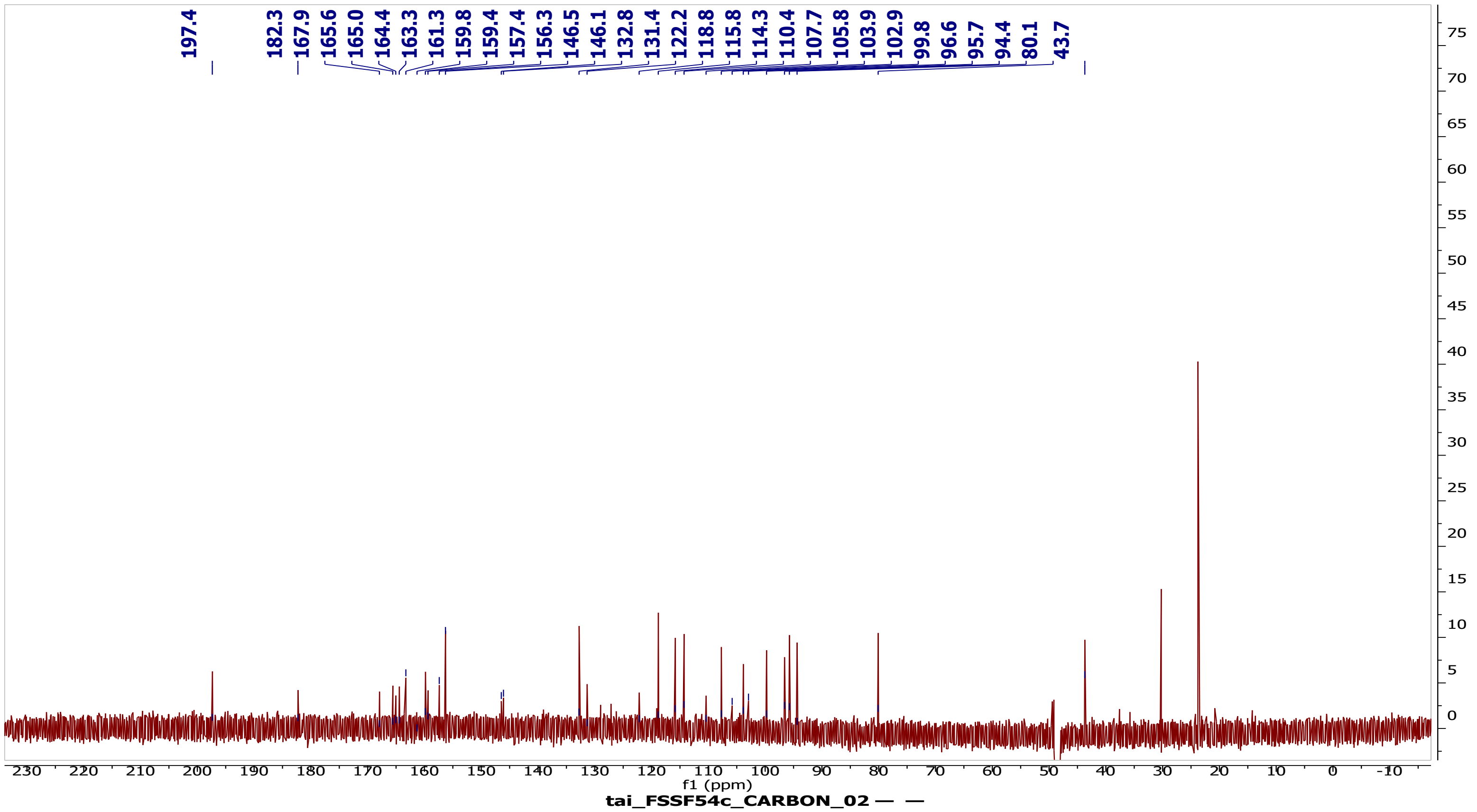

Figure S-2. ^{13}C-NMR spectrum of compounds **1** and **2** (150 MHz, CD_3OD)

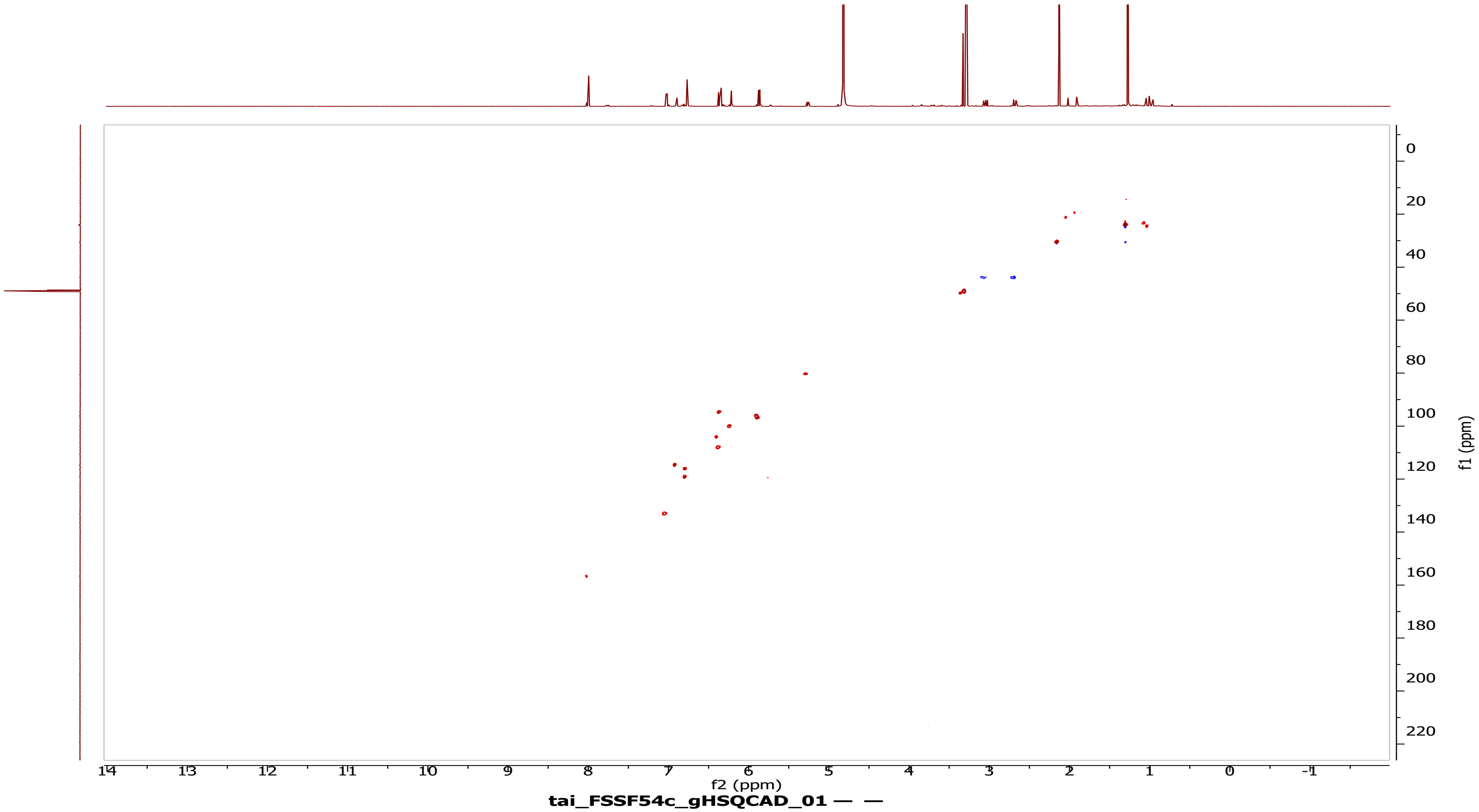

Figure S-3. HSQC spectrum of compounds **1** and **2**

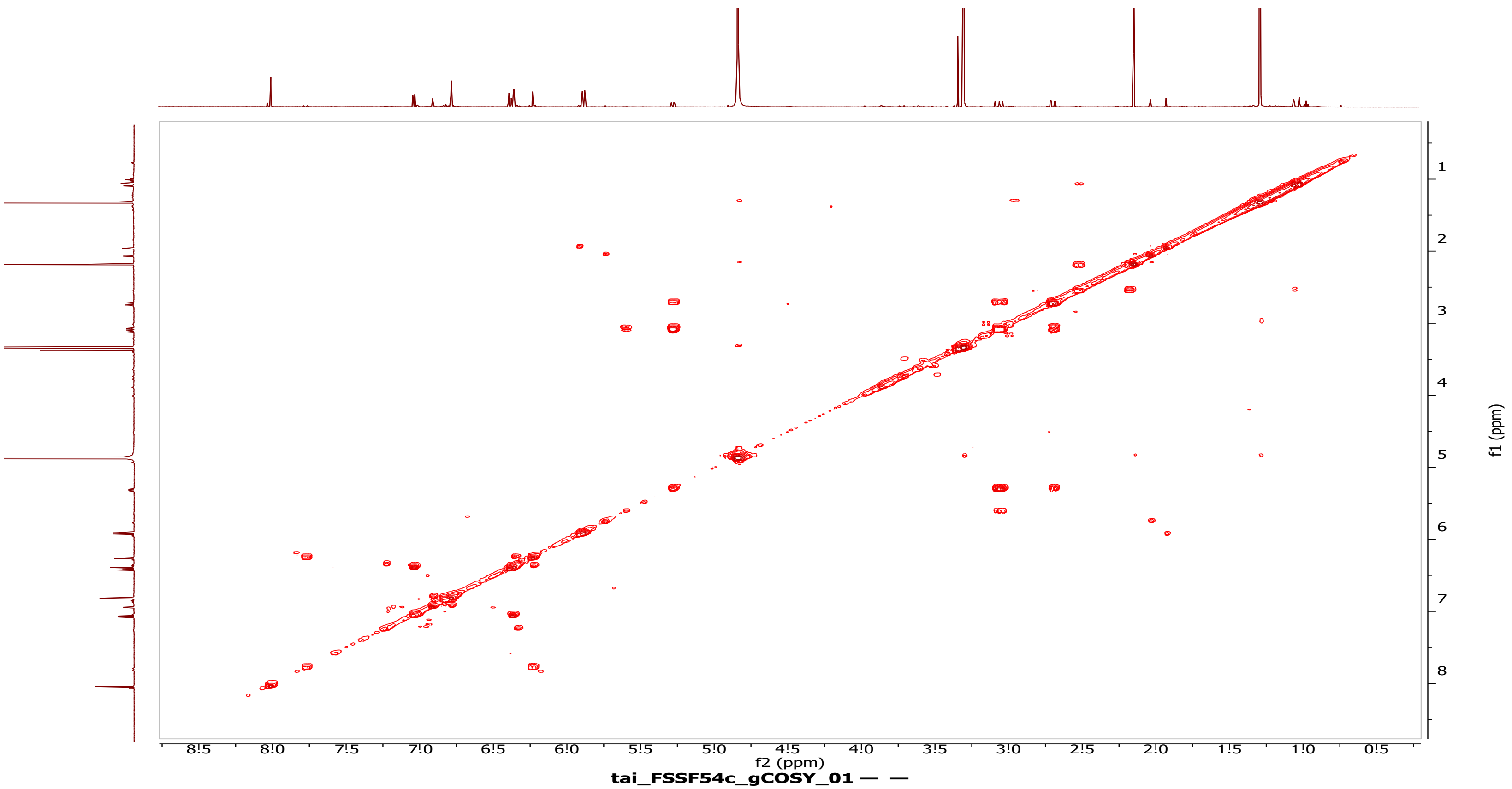

Figure S-4. COSY spectrum of compounds **1** and **2**

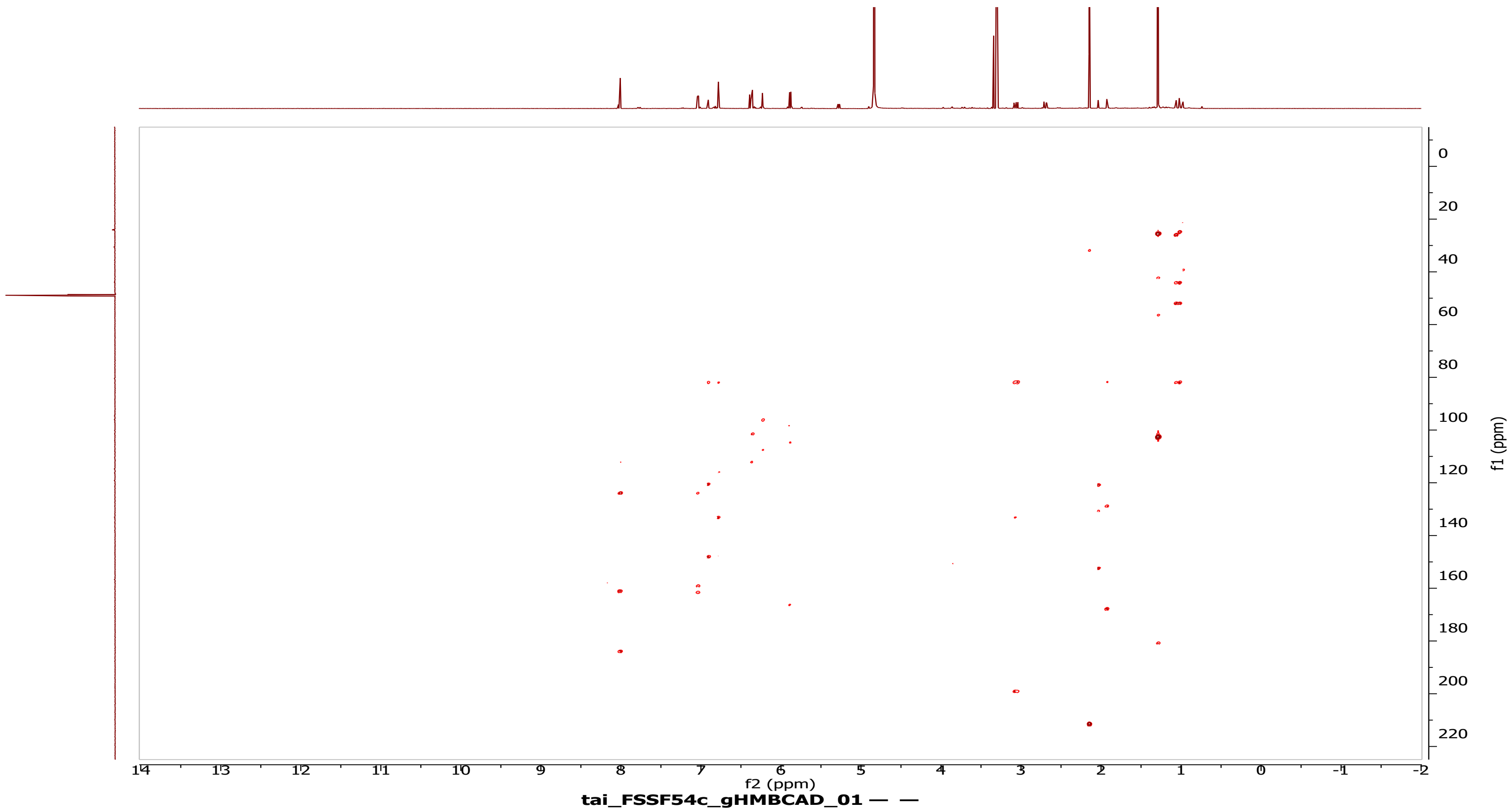

Figure S-5. HMBC spectrum of compounds **1** and **2**

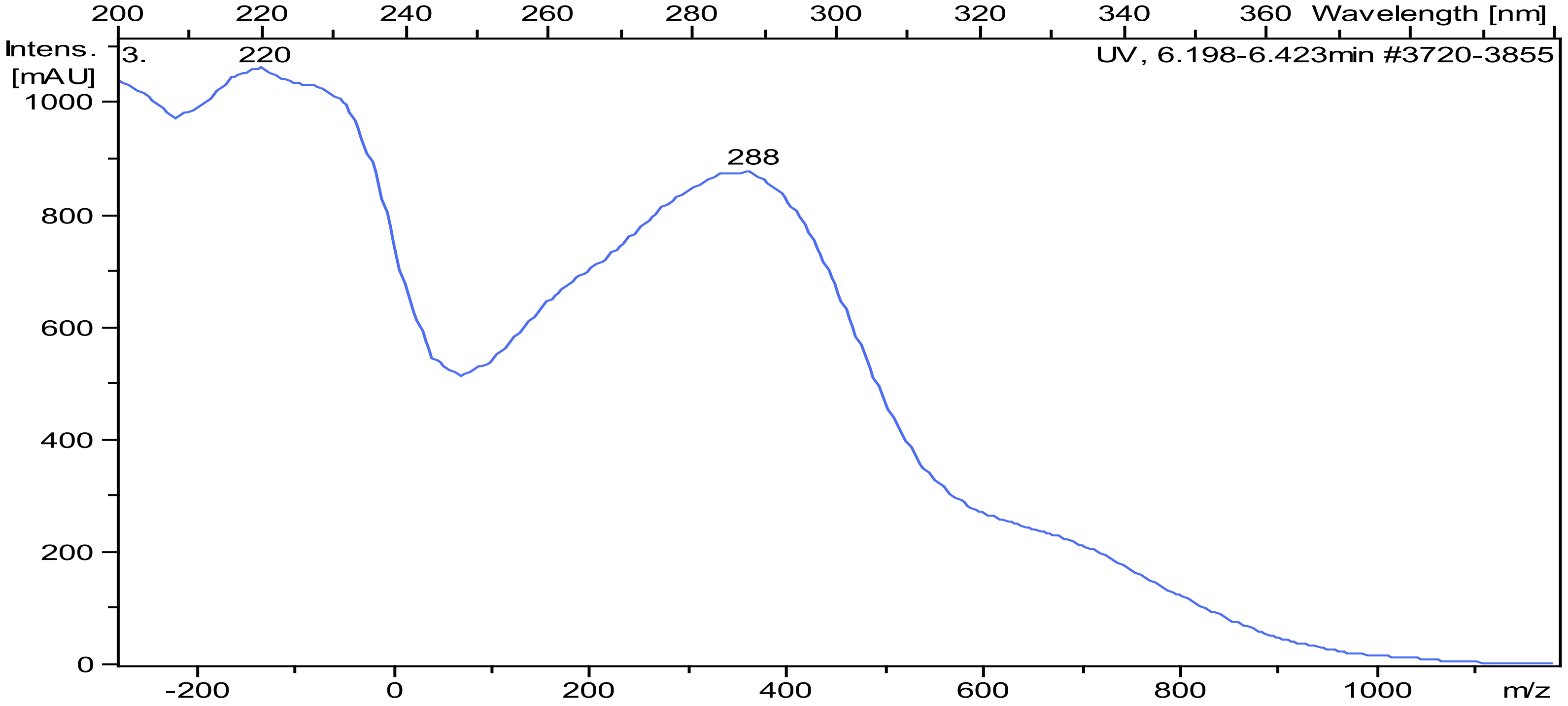

Figure S-6. LC-UV spectrum of compound **1**

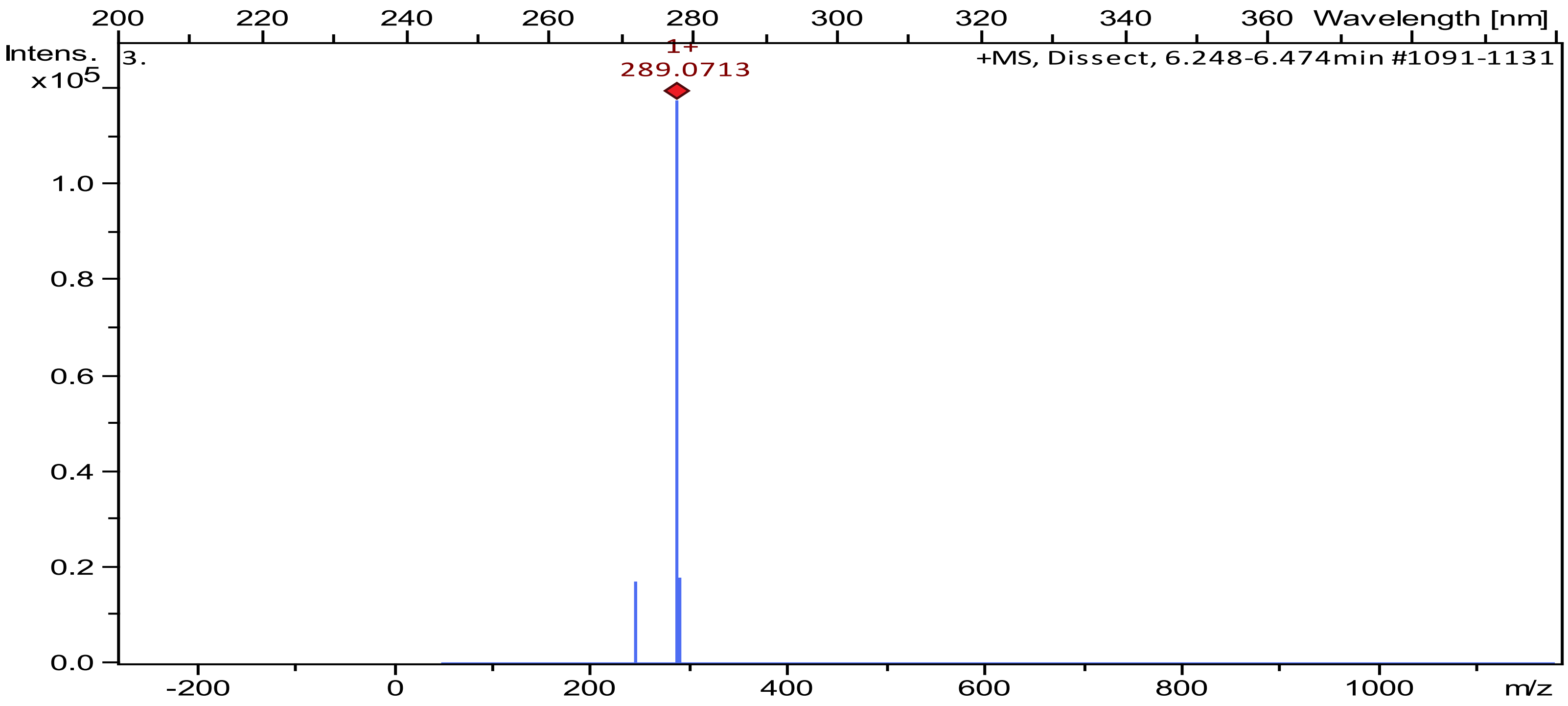

Figure S-7. HR-ESIMS spectrum of compound **1**

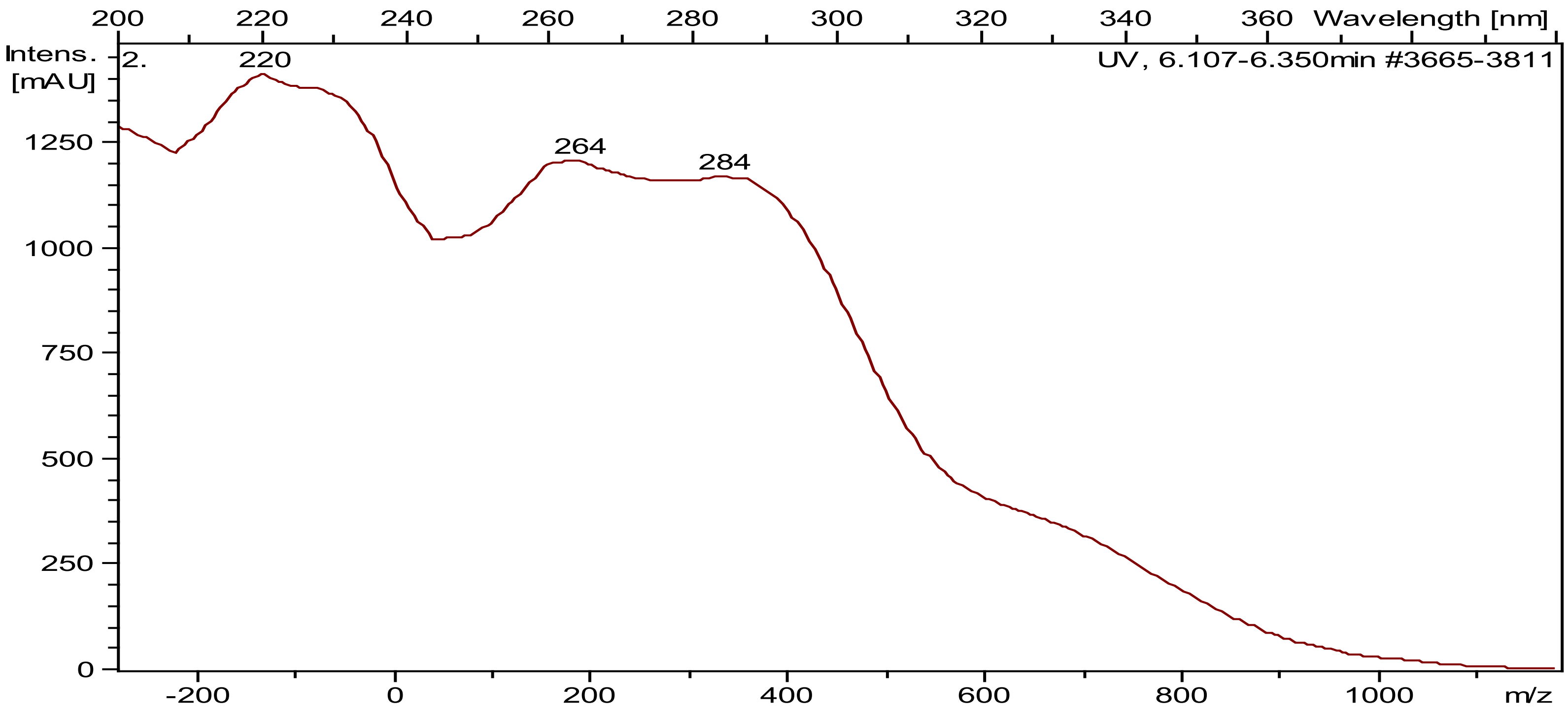

Figure S-8. LC-UV spectrum of compound **2**

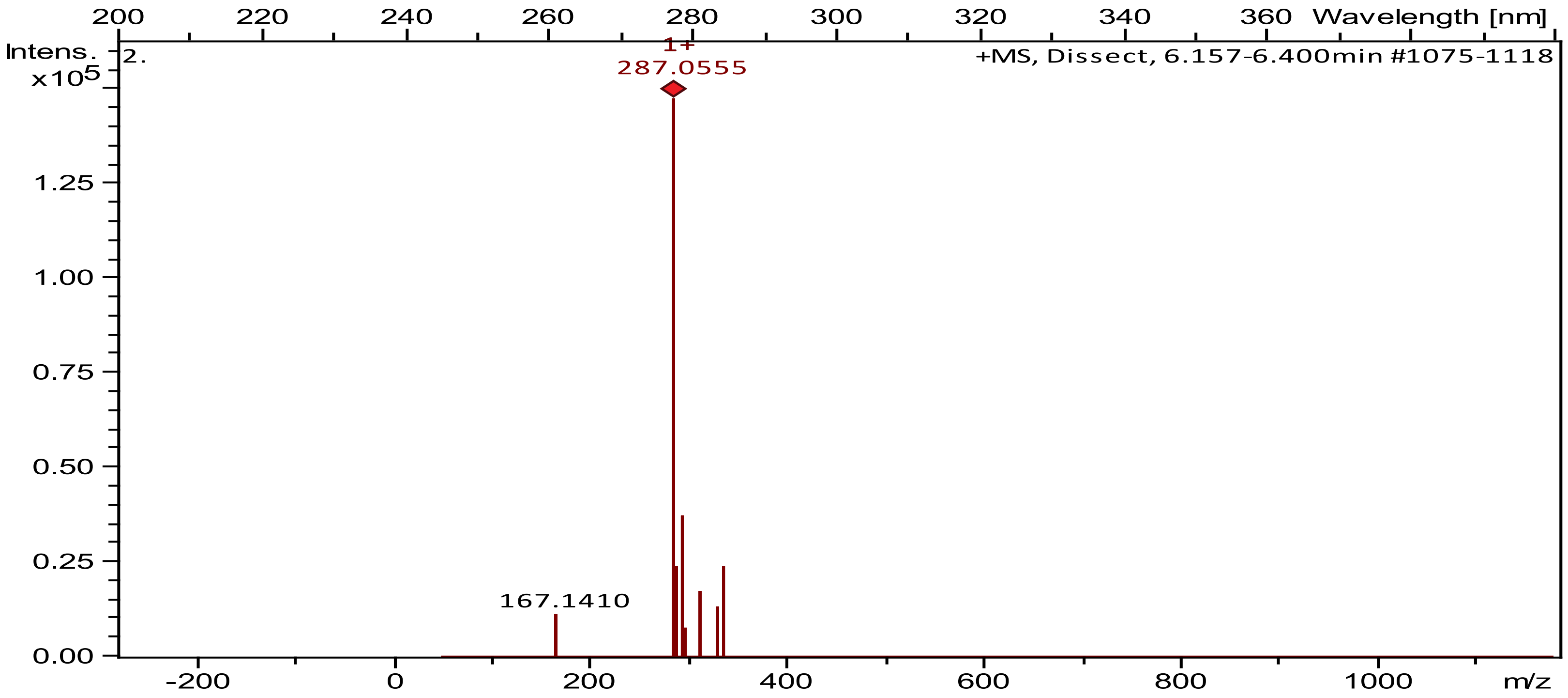

Figure S-9. HR-ESIMS spectrum of compound **2**

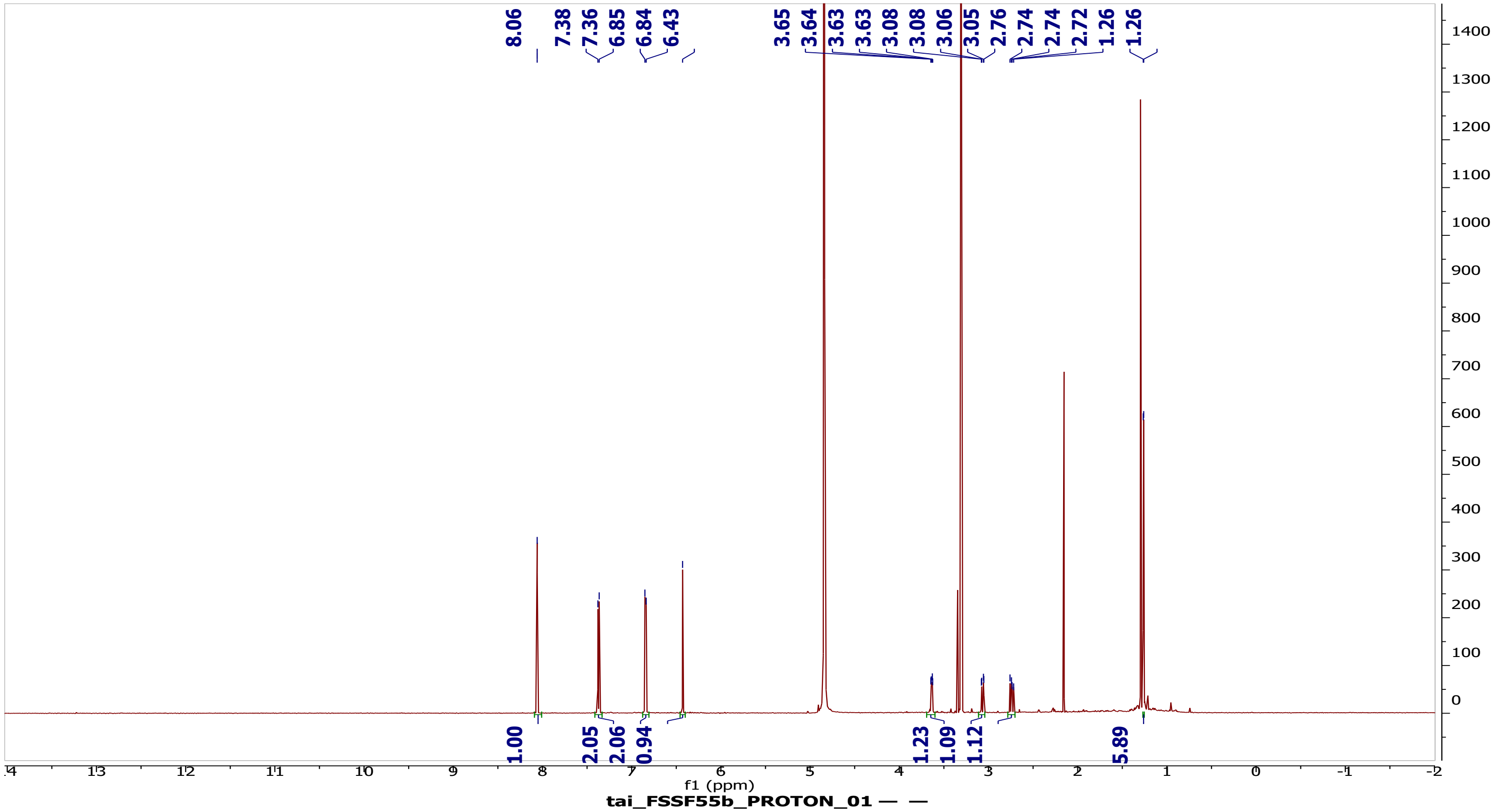

Figure S-10. ^{1}H-NMR spectrum of compound **3** (600 MHz, CD_3OD)

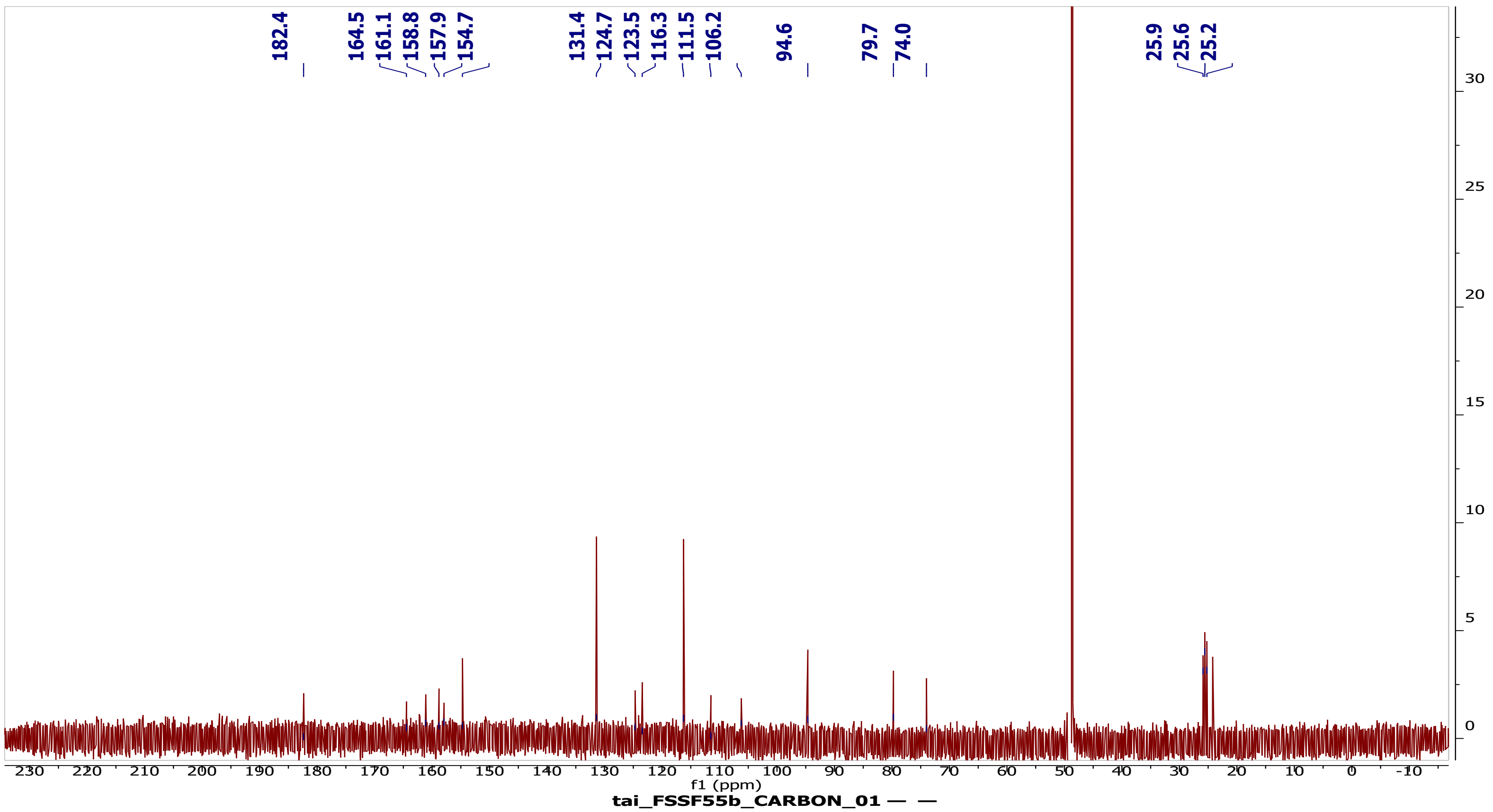

Figure S-11. ^{13}C-NMR spectrum of compound **3** (150 MHz, CD_3OD)

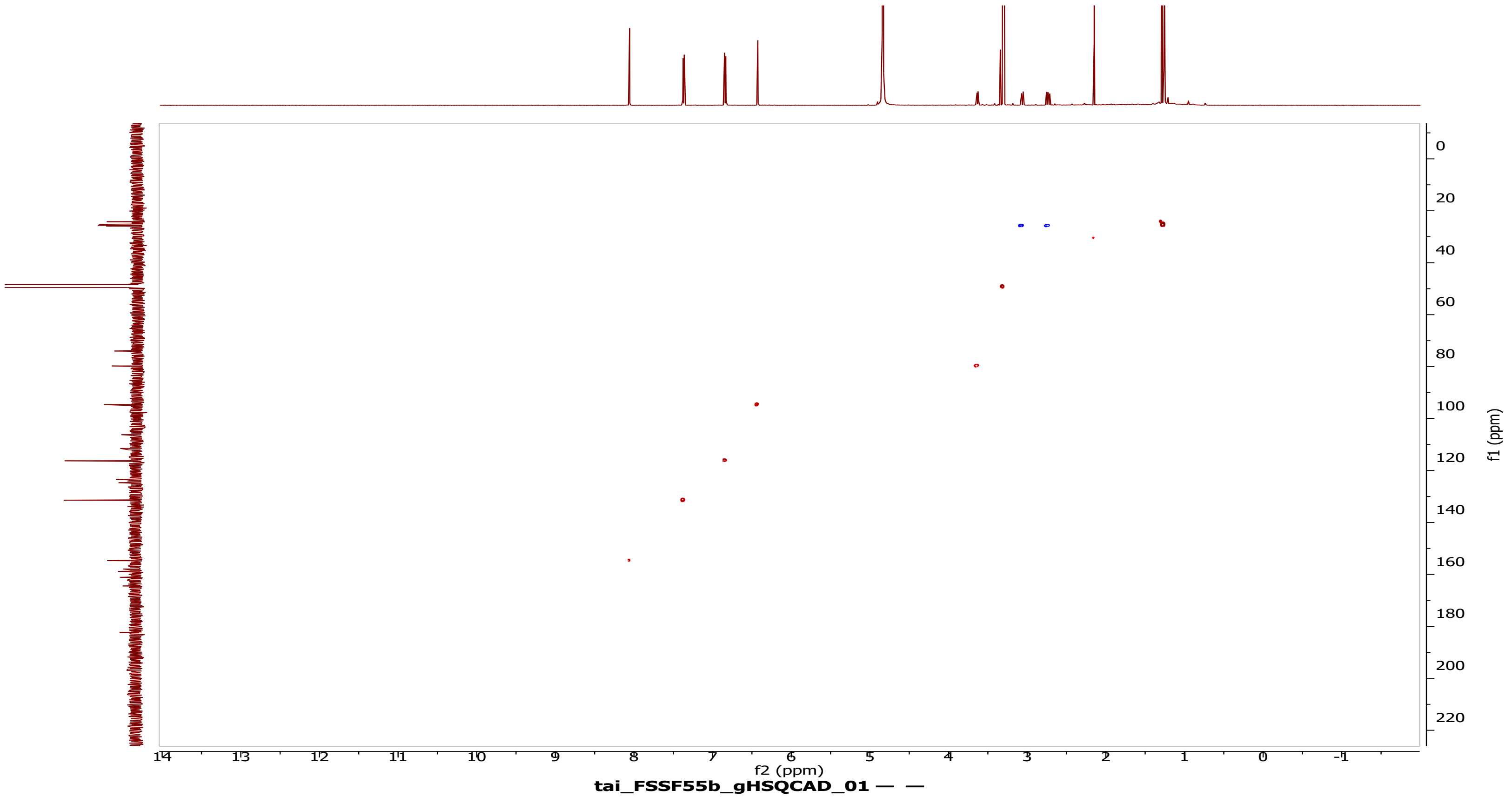

Figure S-12. HSQC spectrum of compound **3**

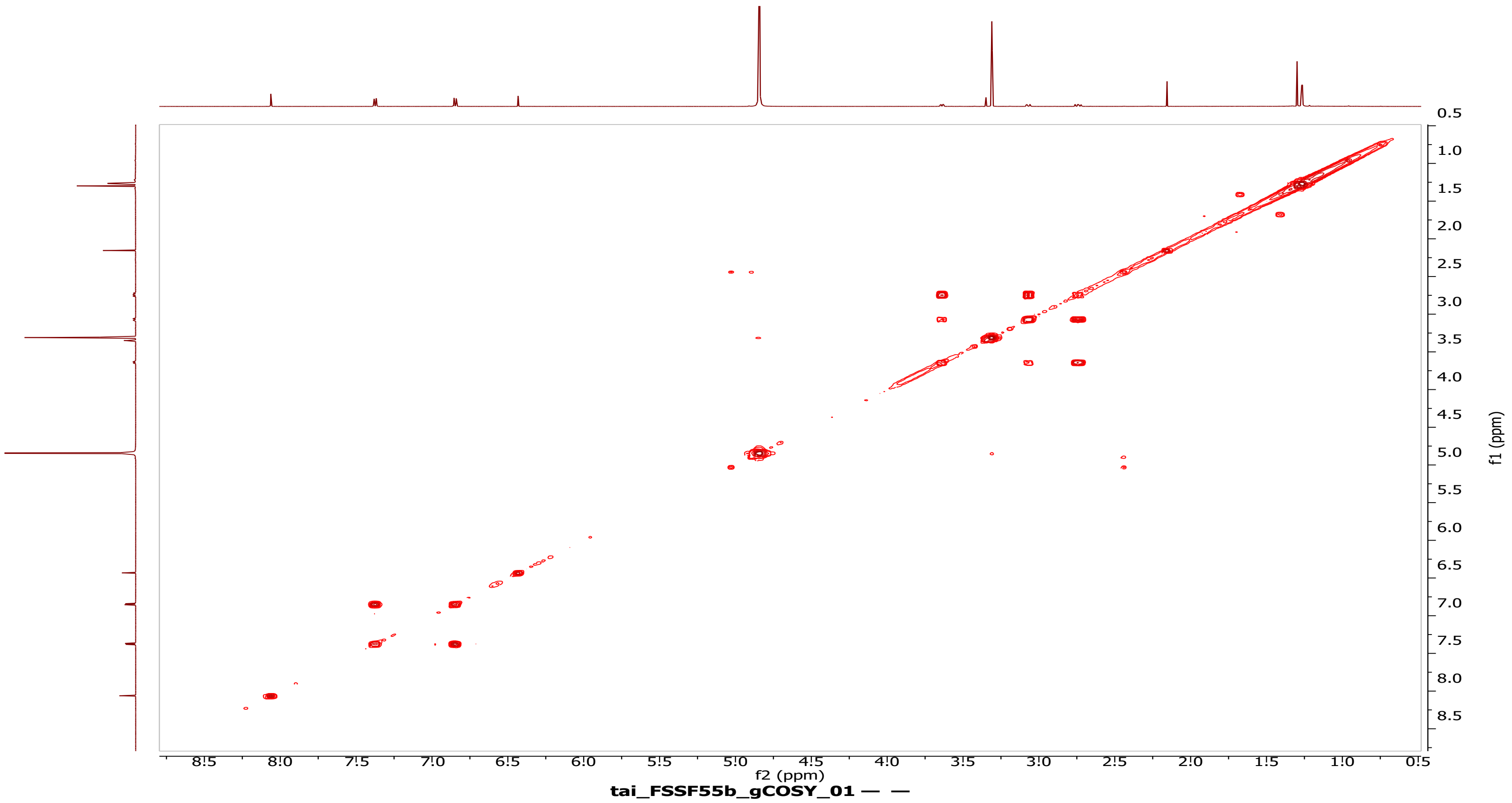

Figure S-13. COSY spectrum of compound **3**

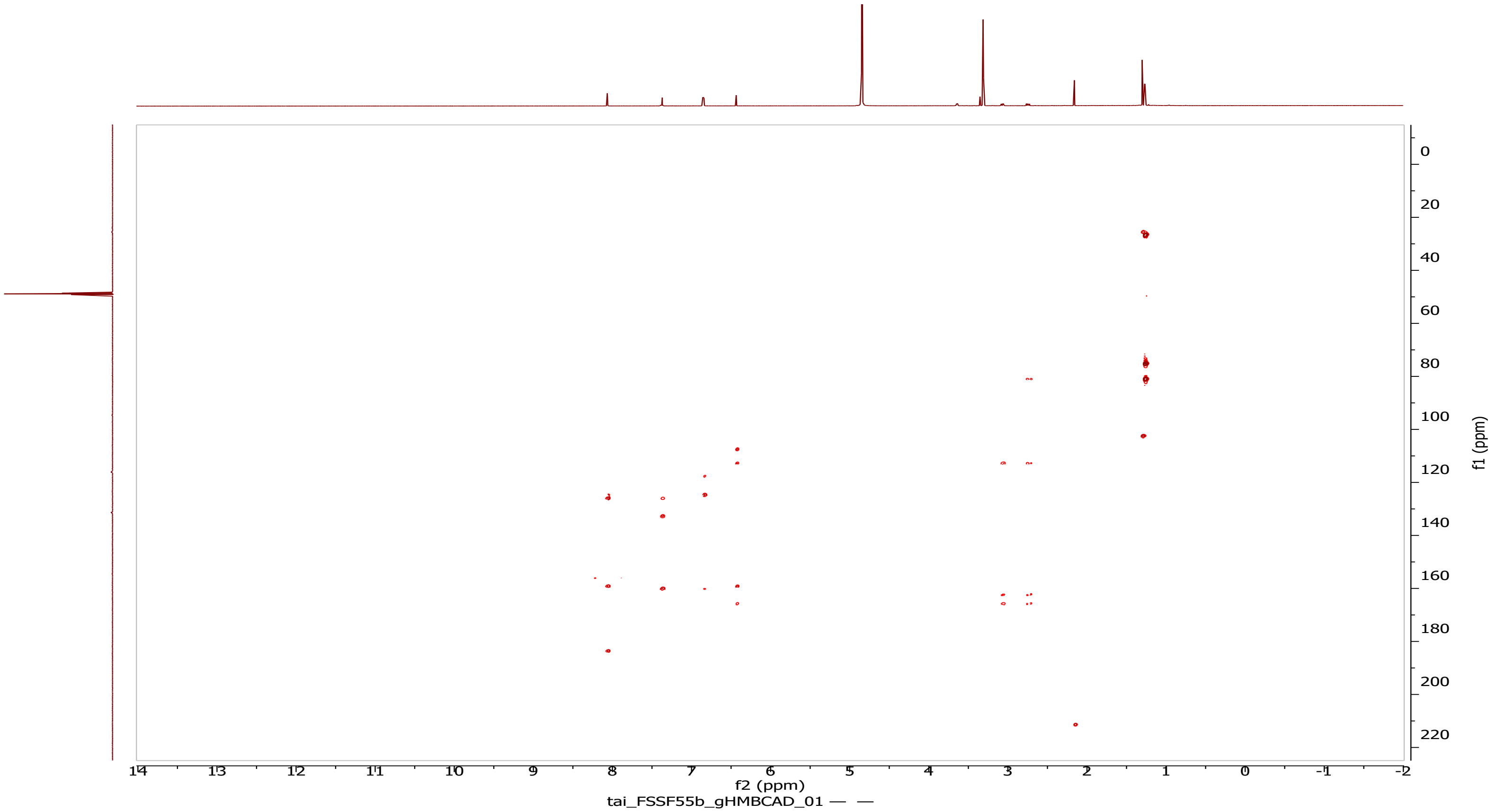

Figure S-14. HMBC spectrum of compound **3**

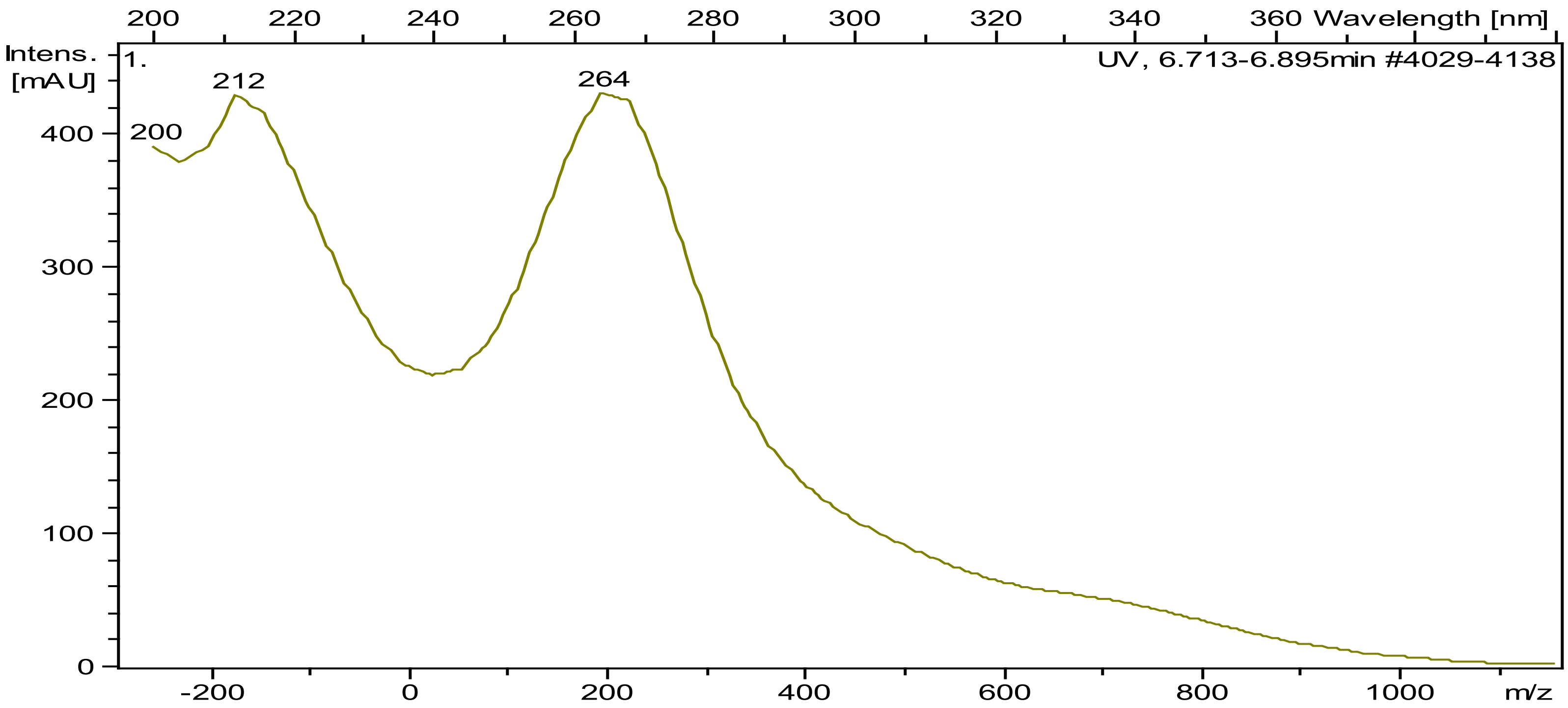

Figure S-15. LC-UV spectrum of compound **3**

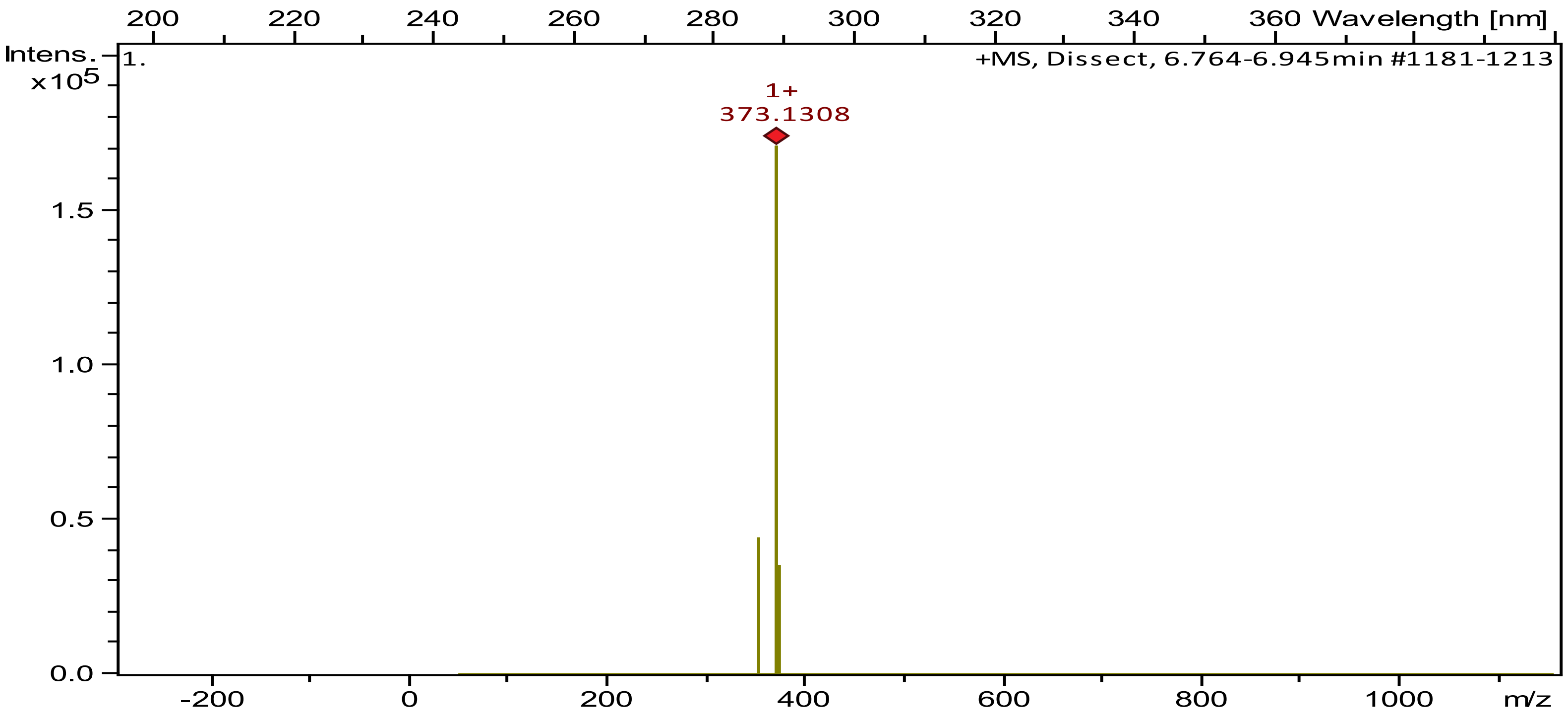

Figure S-16. HR-ESIMS spectrum of compound **3**

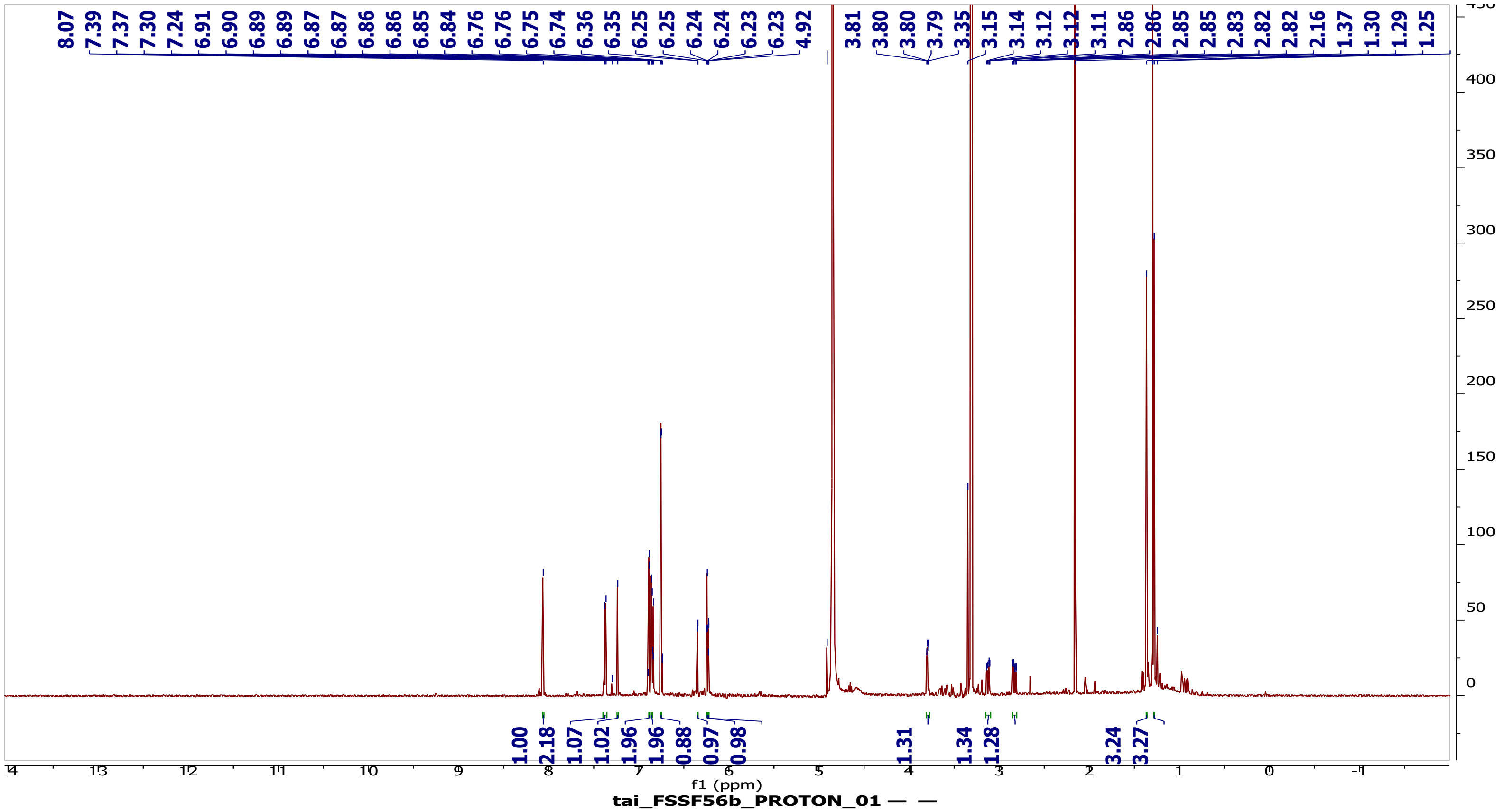

Figure S-17. ^{1}H-NMR spectrum of compounds **4** and **5** (600 MHz, CD_3OD)

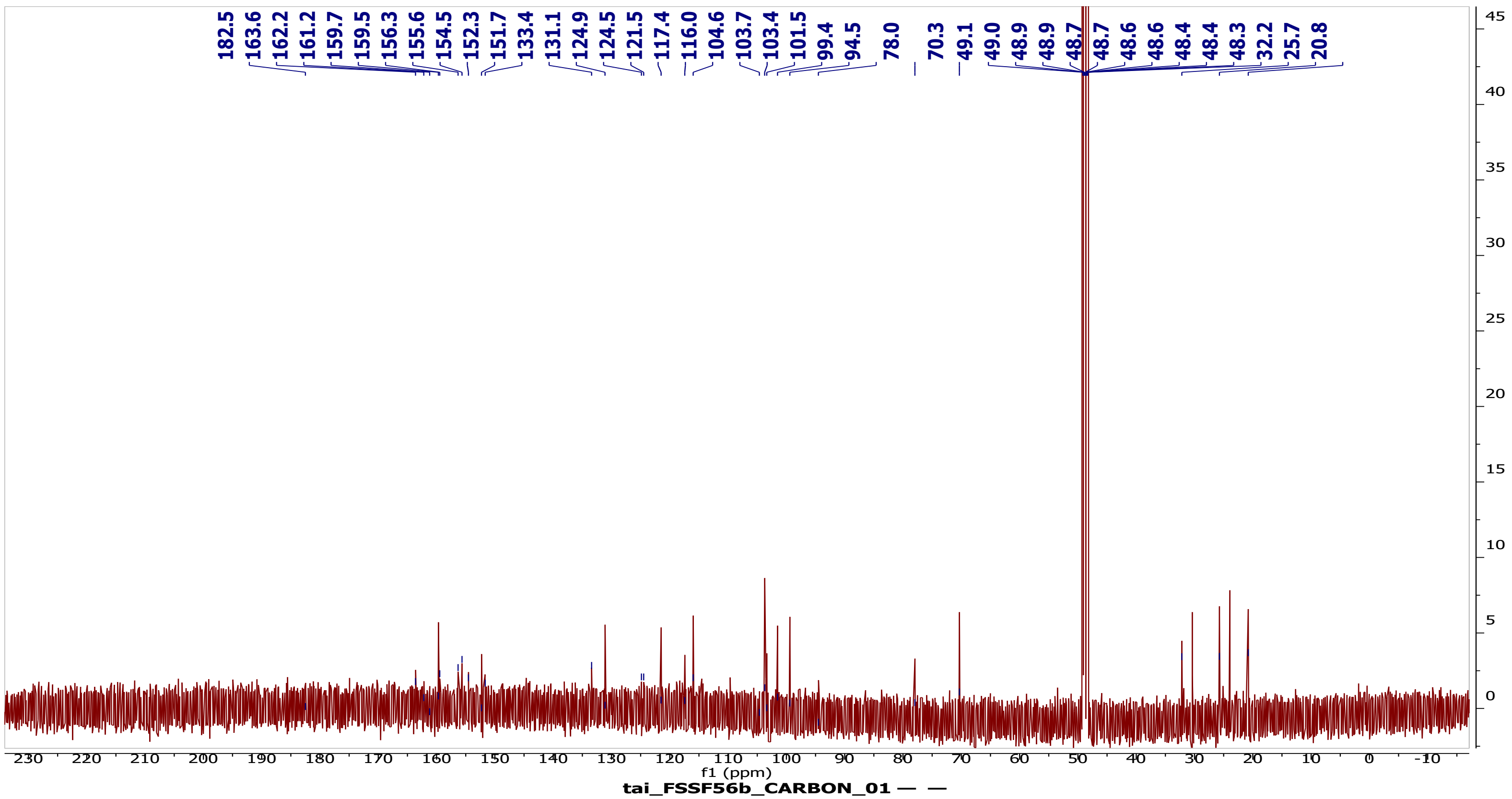

Figure S-18. ^{13}C-NMR spectrum of compounds **4** and **5** (150 MHz, CD_3OD)

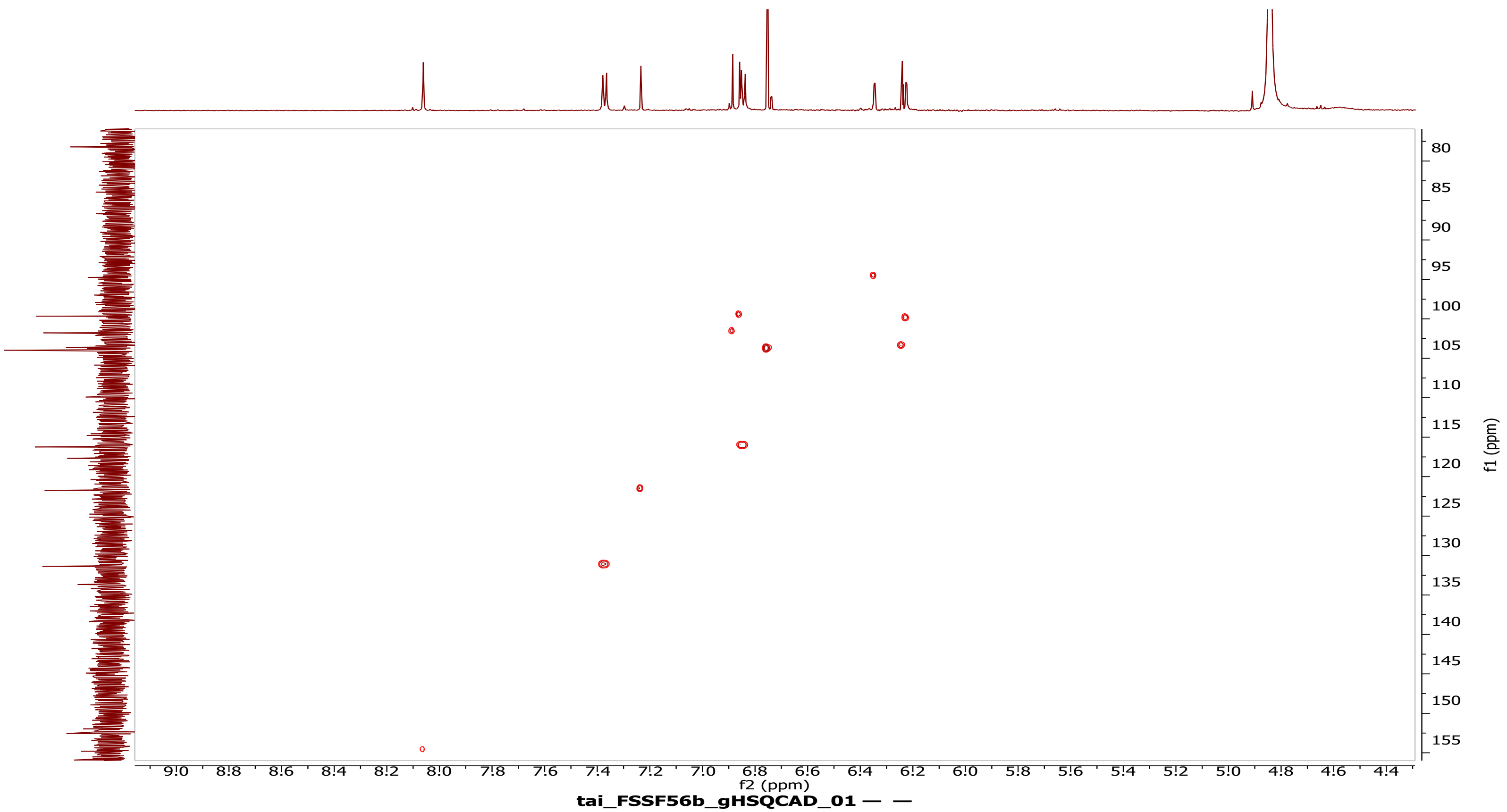

Figure S-19. HSQC spectrum of compounds **4** and **5**

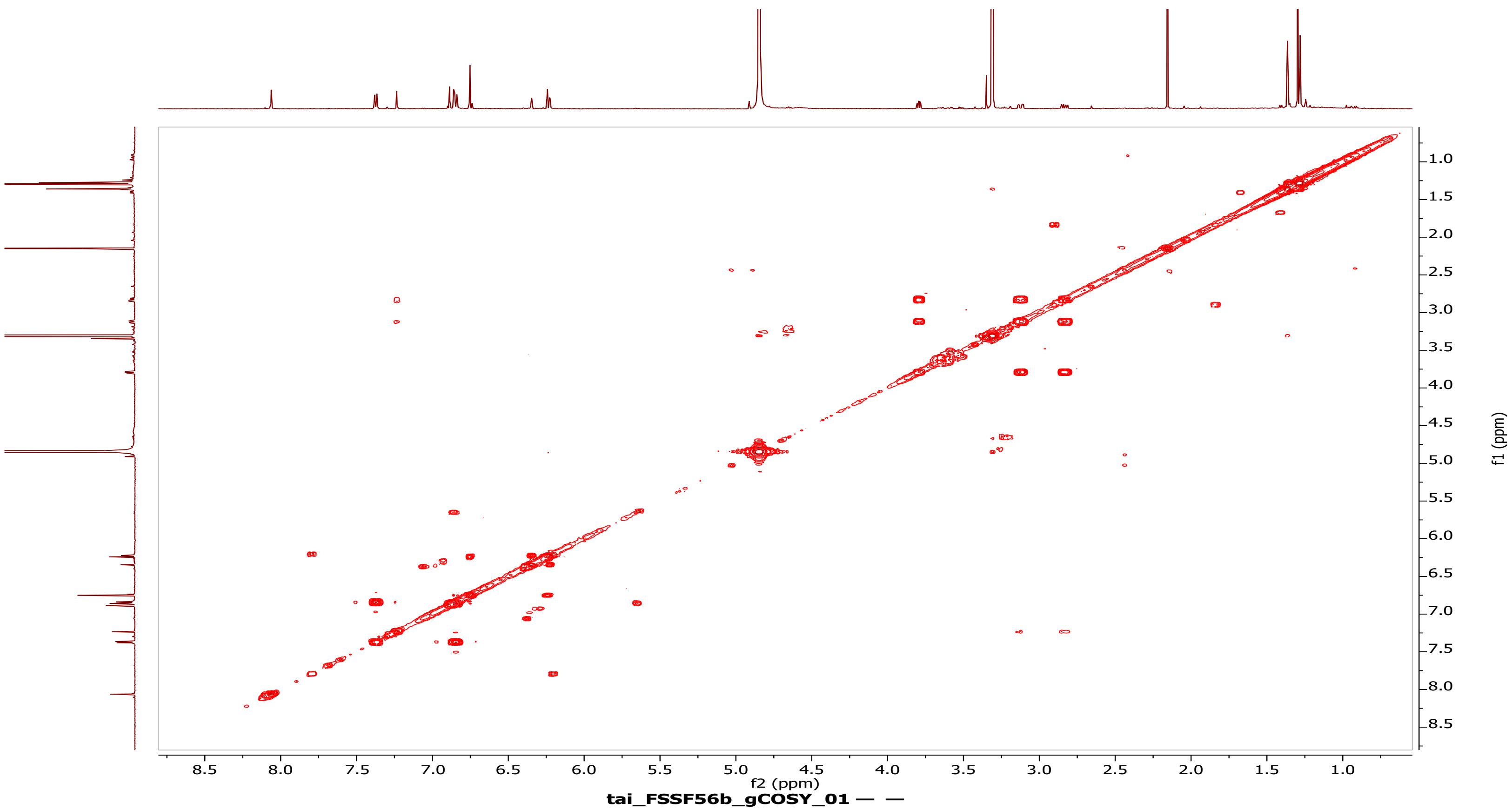

Figure S-20. COSY spectrum of compounds **4** and **5**

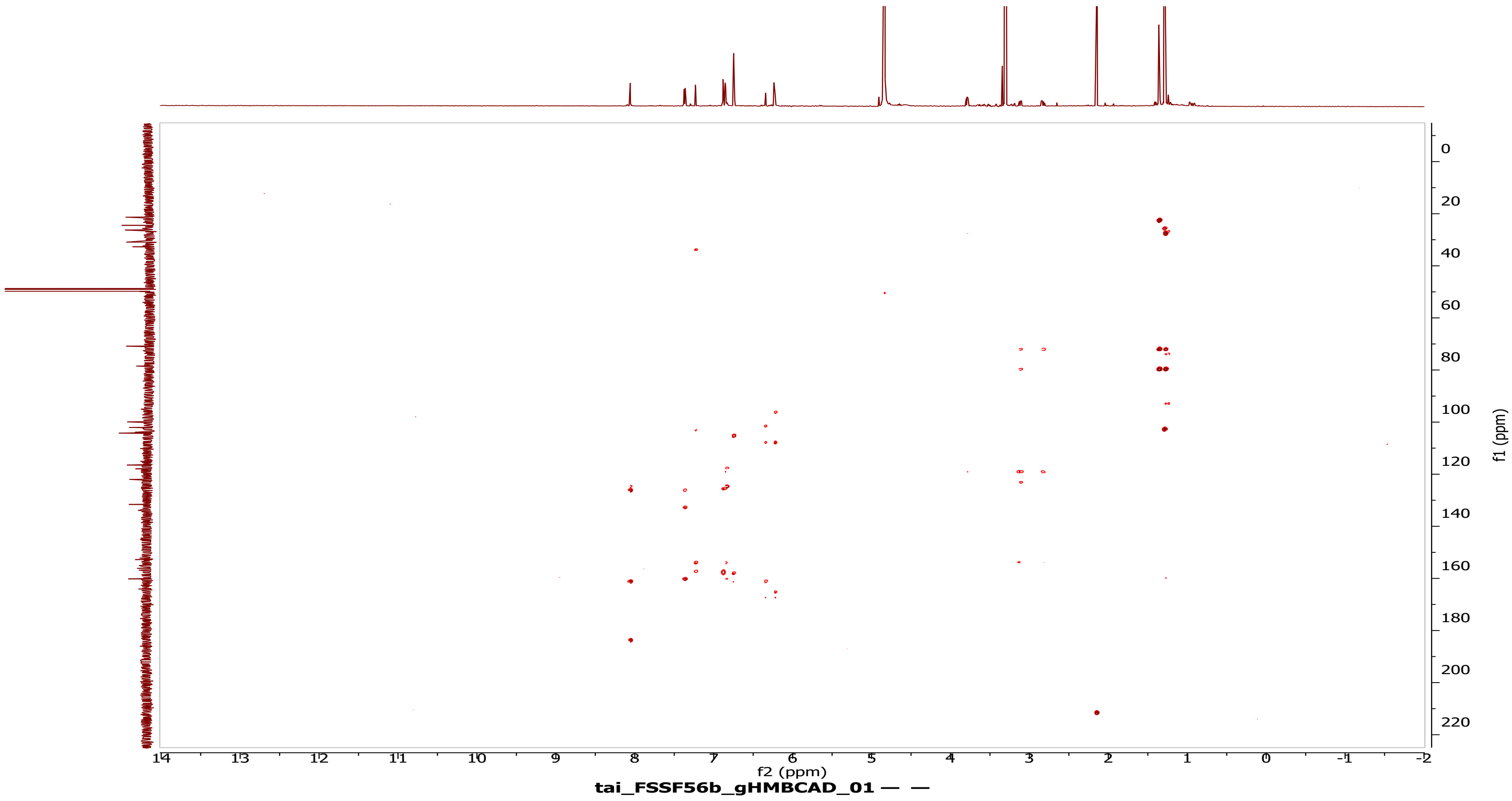

Figure S-21. HMBC spectrum of compounds **4** and **5**

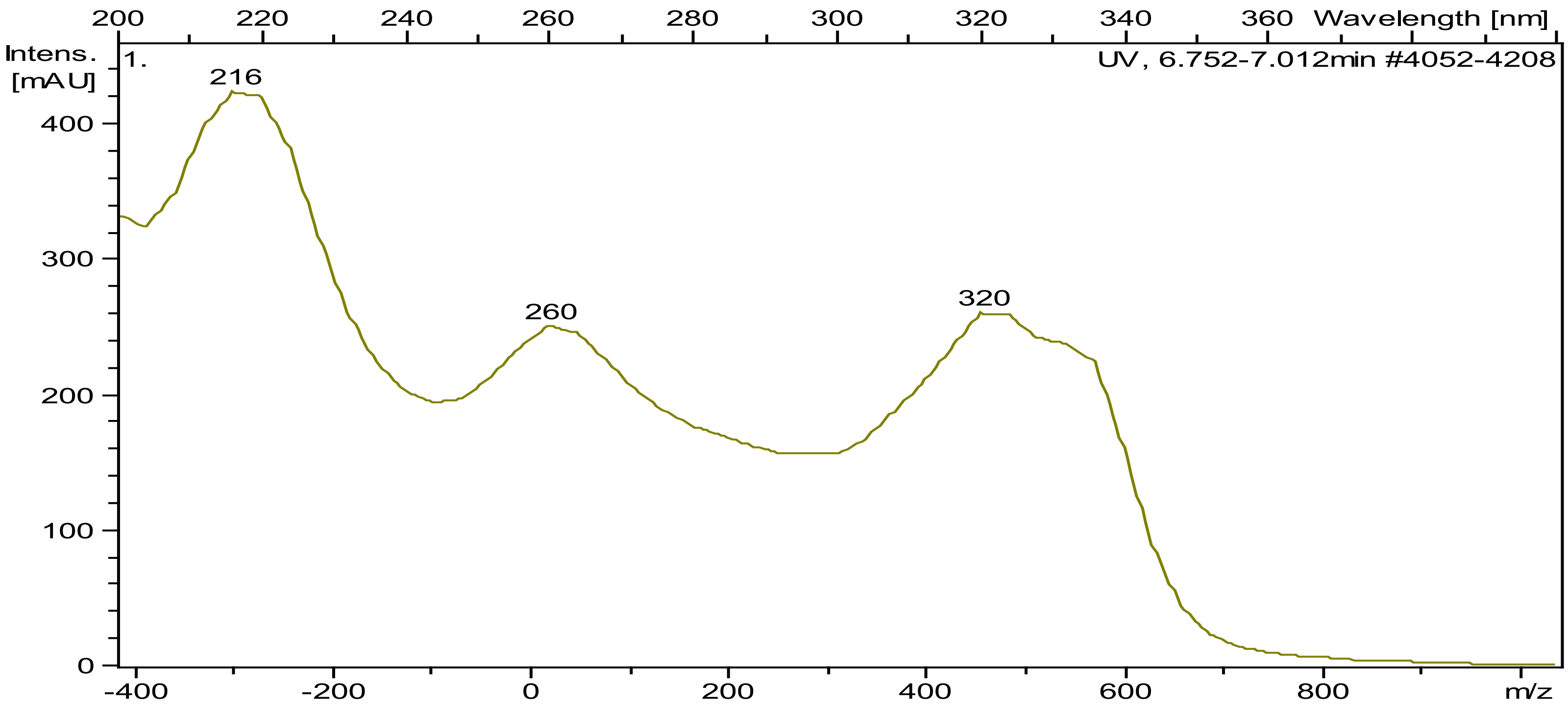

Figure S-22. LC-UV spectrum of compound **4**

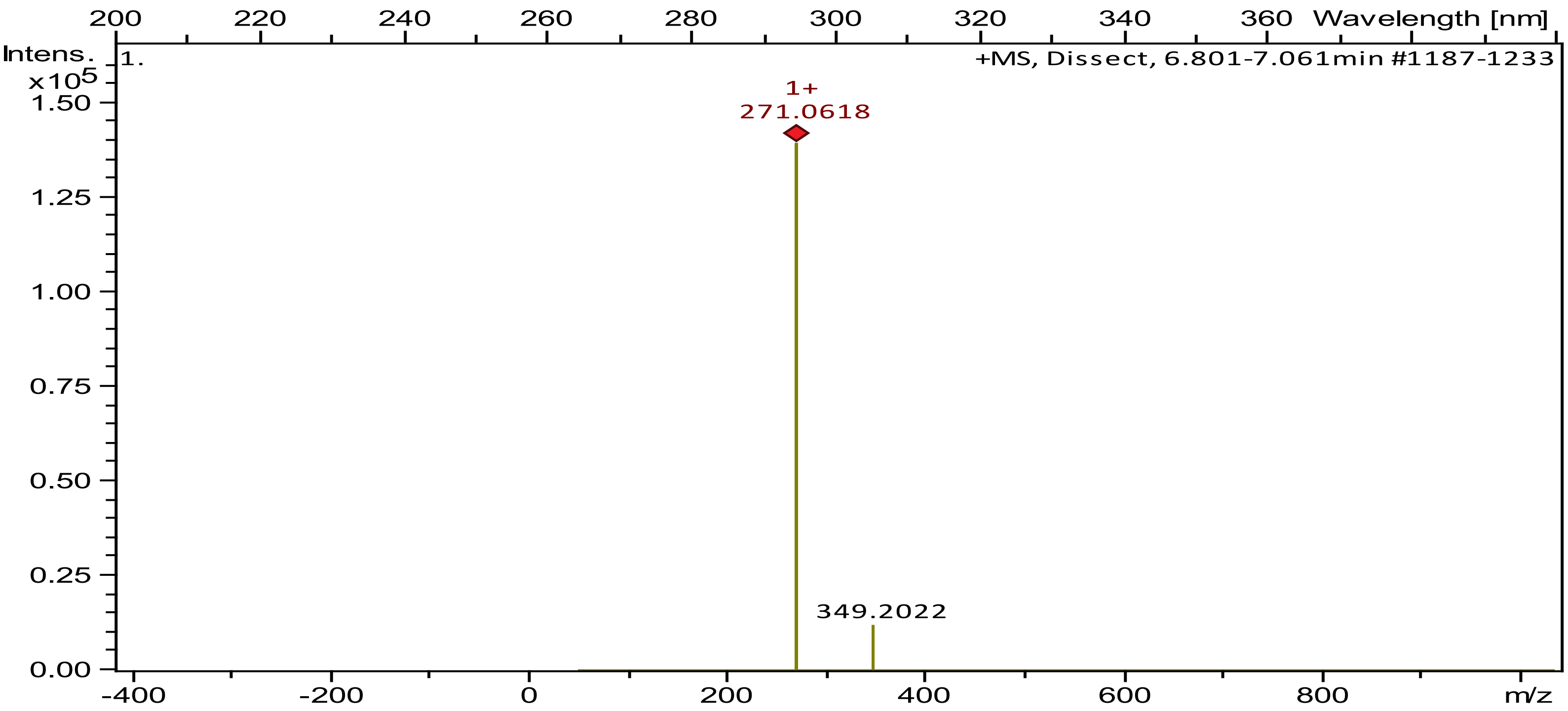

Figure S-23. HR-ESIMS spectrum of compound **4**

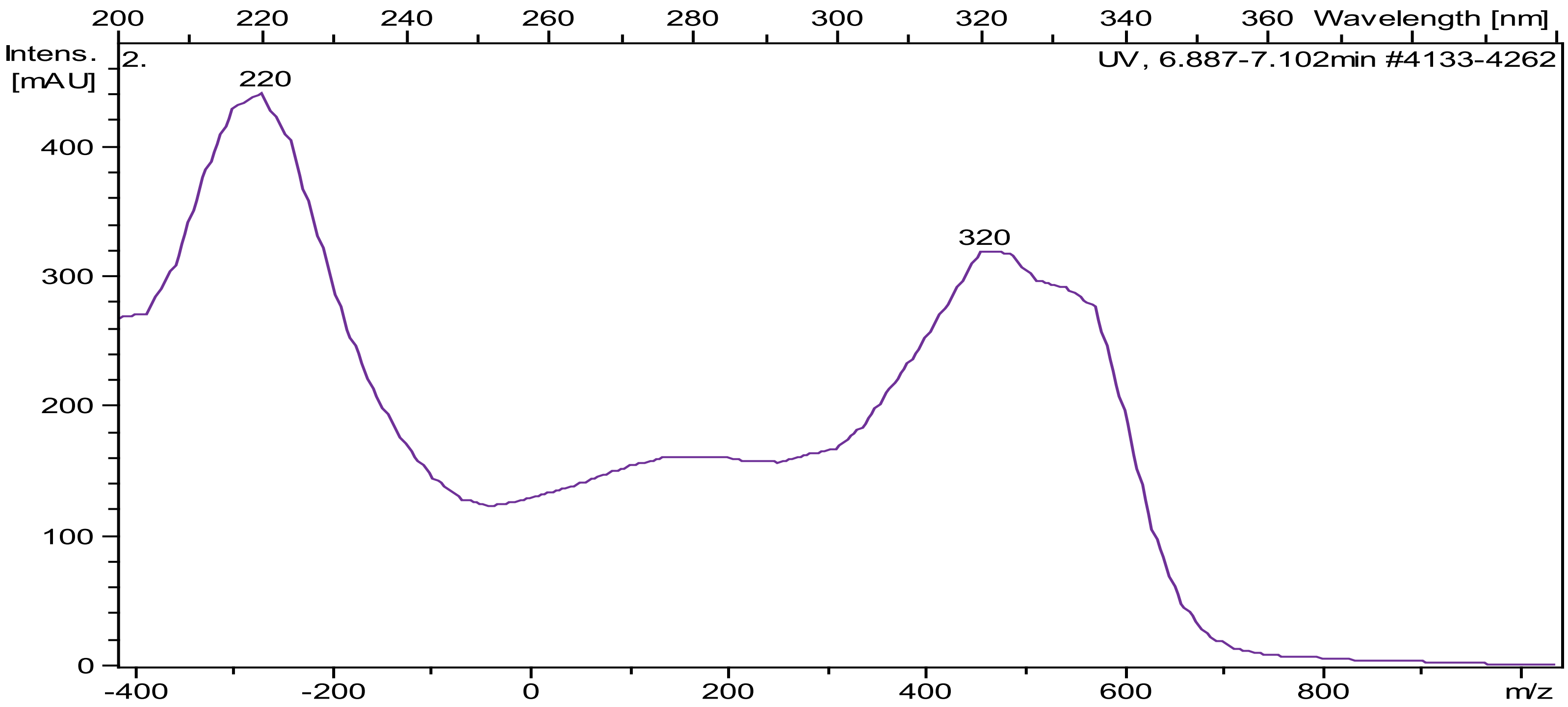

Figure S-24. LC-UV spectrum of compound **5**

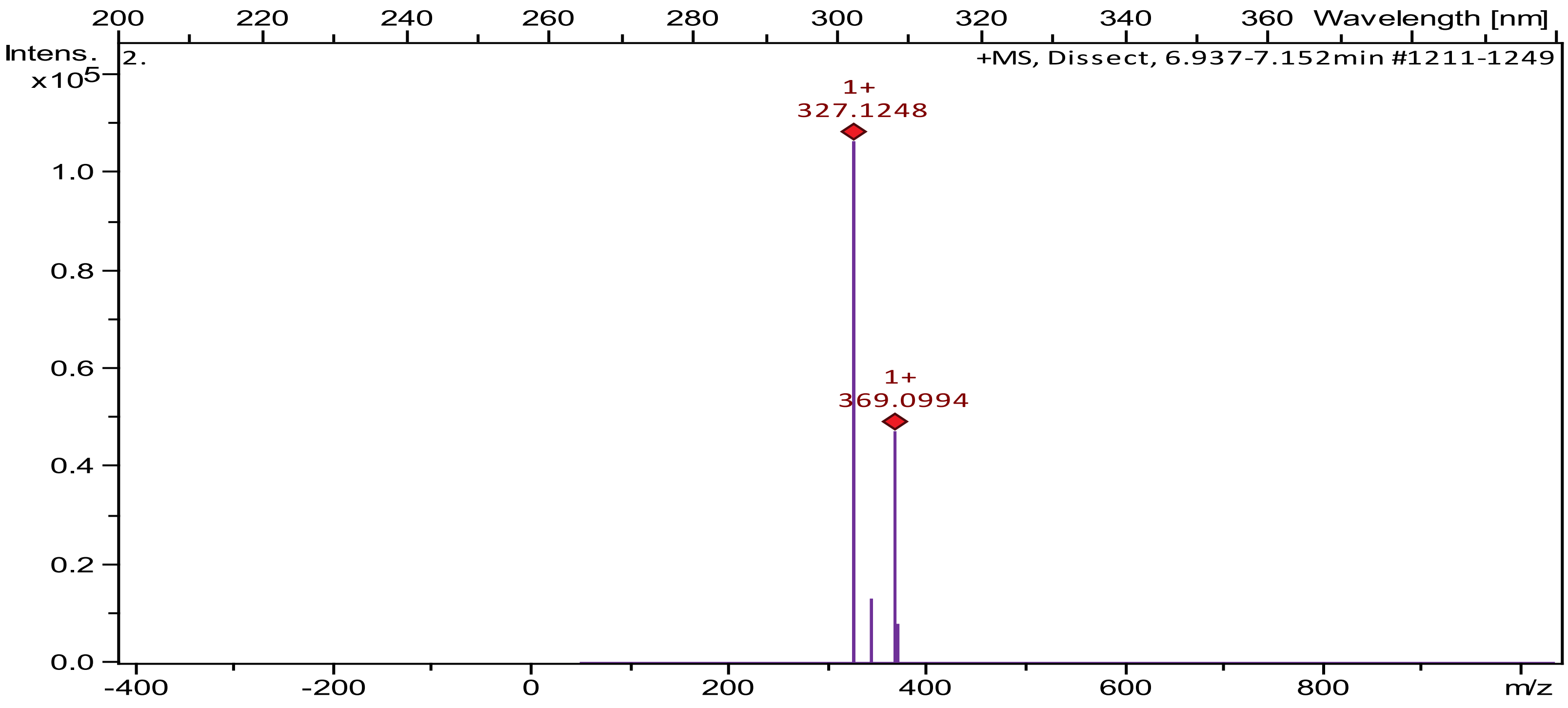

Figure S-25. HR-ESIMS spectrum of compound **5**

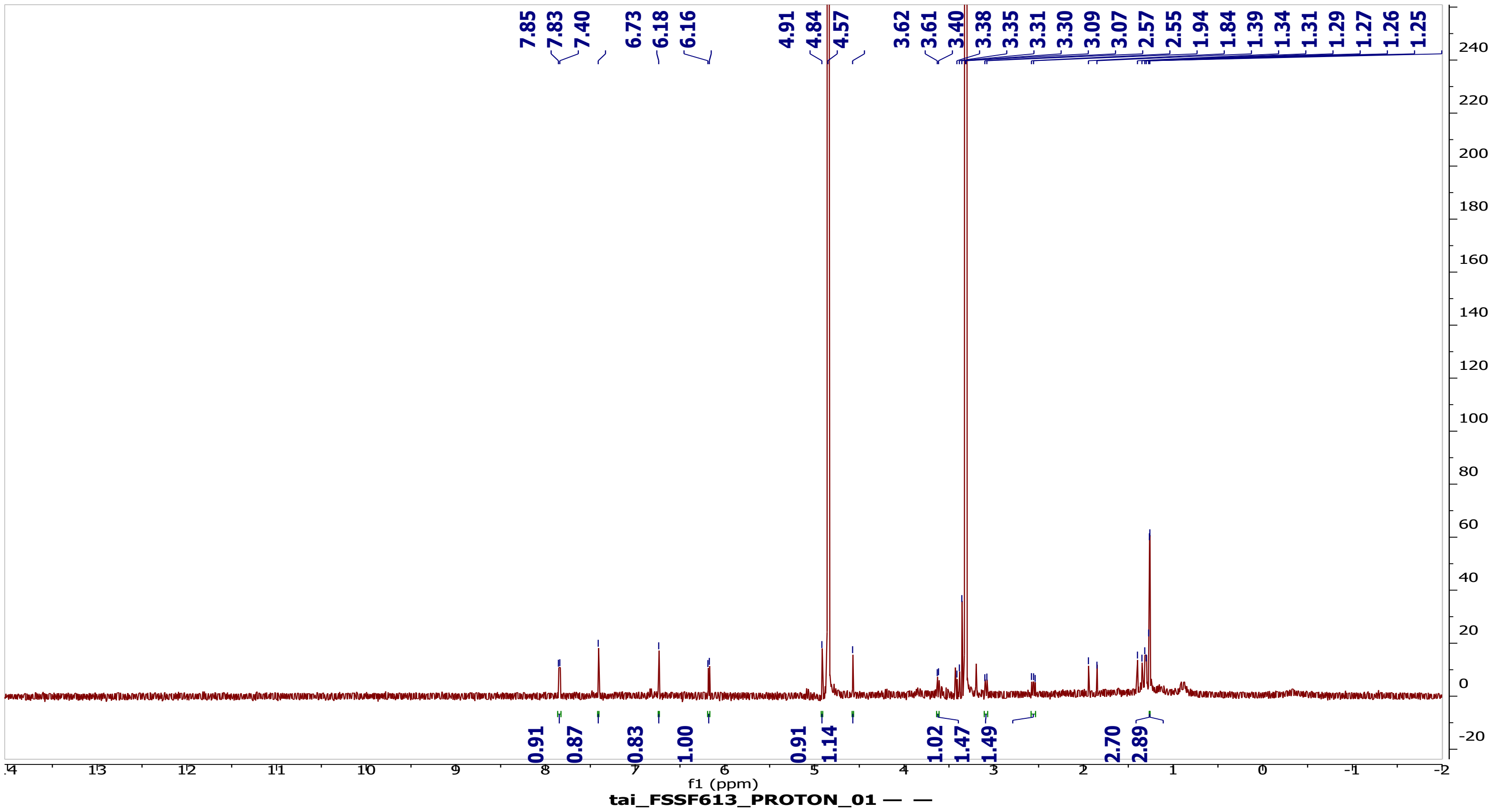

Figure S-26. ^{1}H-NMR spectrum of compound **6** (600 MHz, CD_3OD)

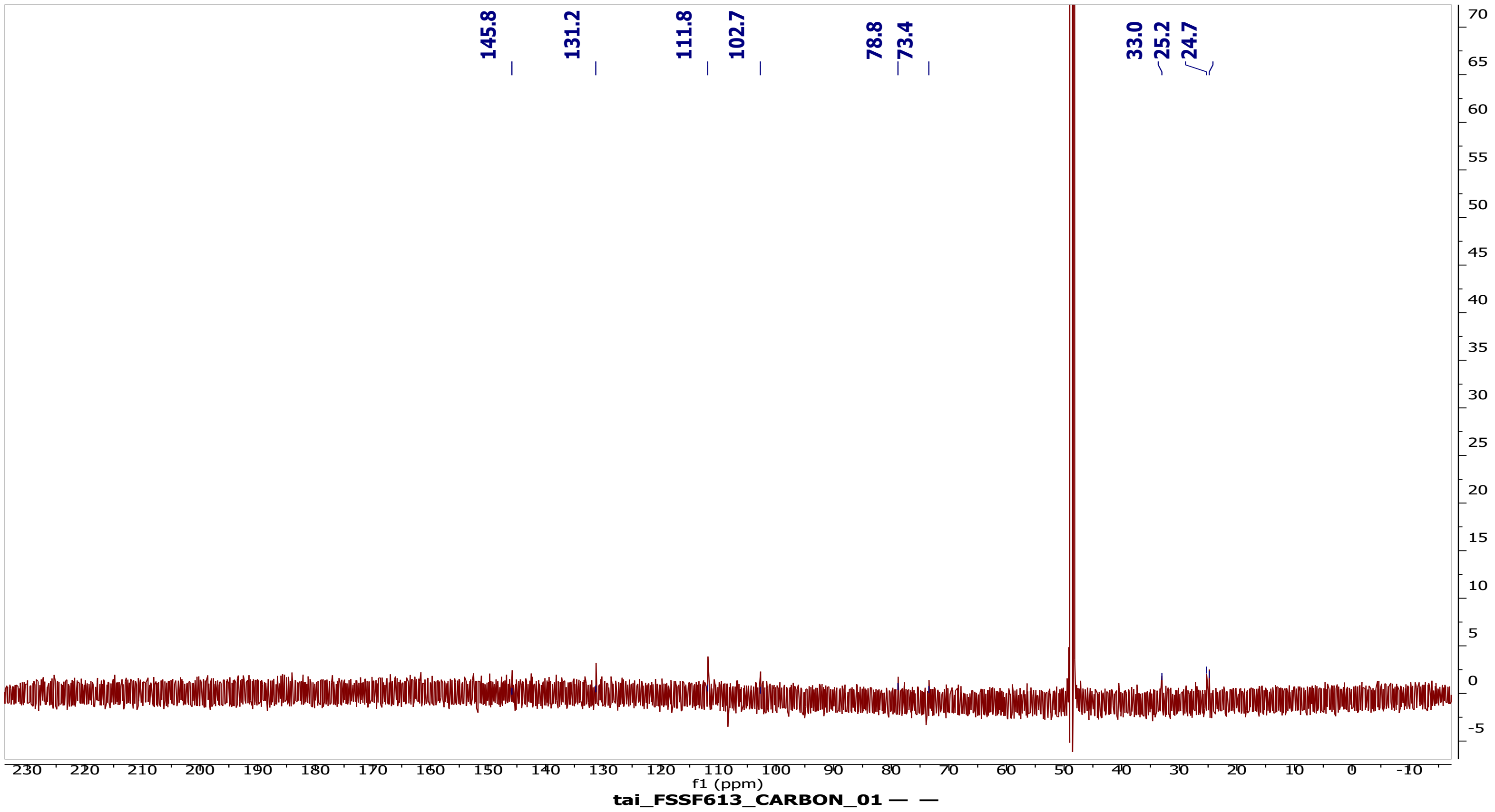

Figure S-27. ^{13}C-NMR spectrum of compound **6** (150 MHz, CD_3OD)

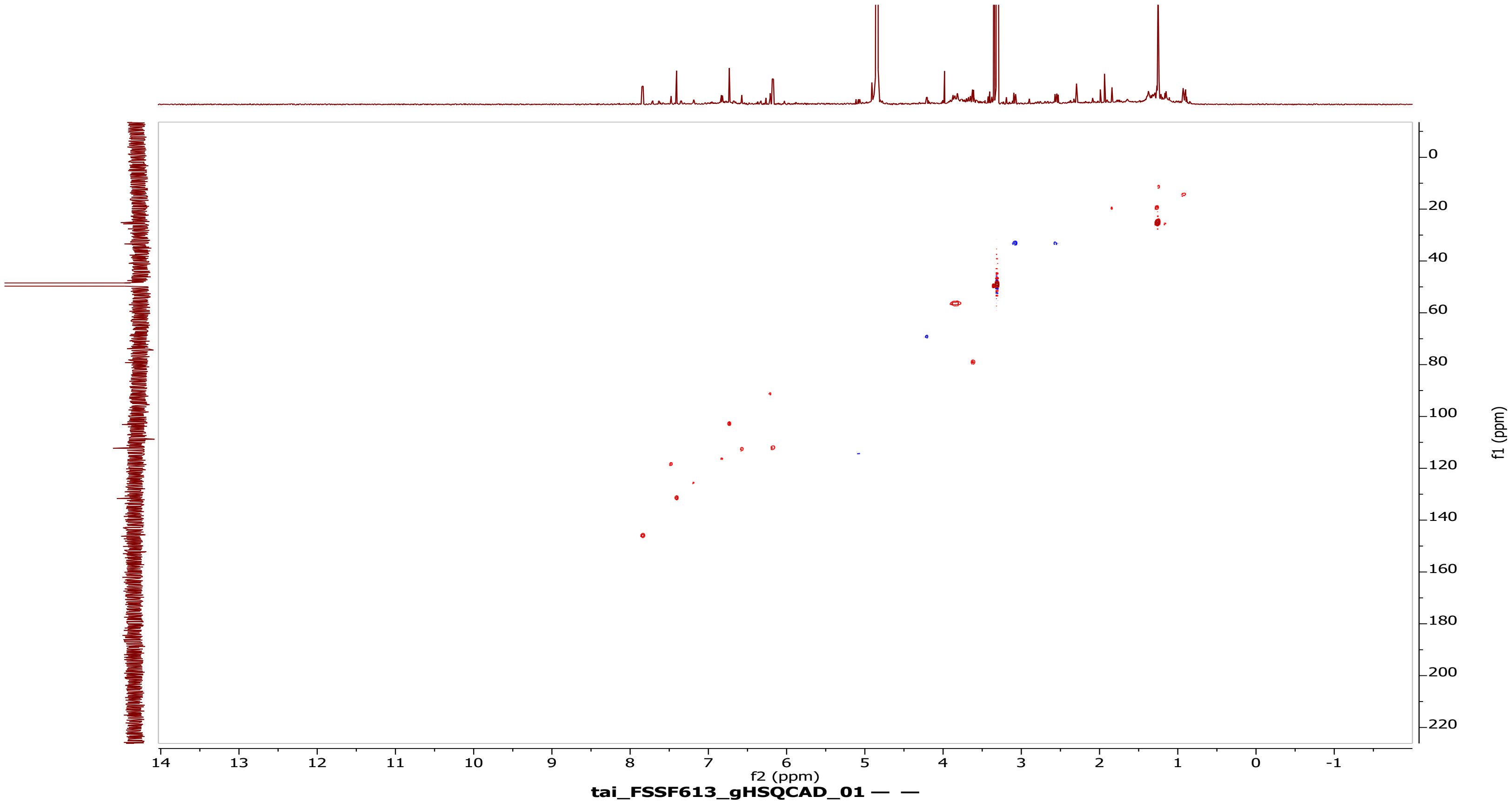

Figure S-28. HSQC spectrum of compound **6**

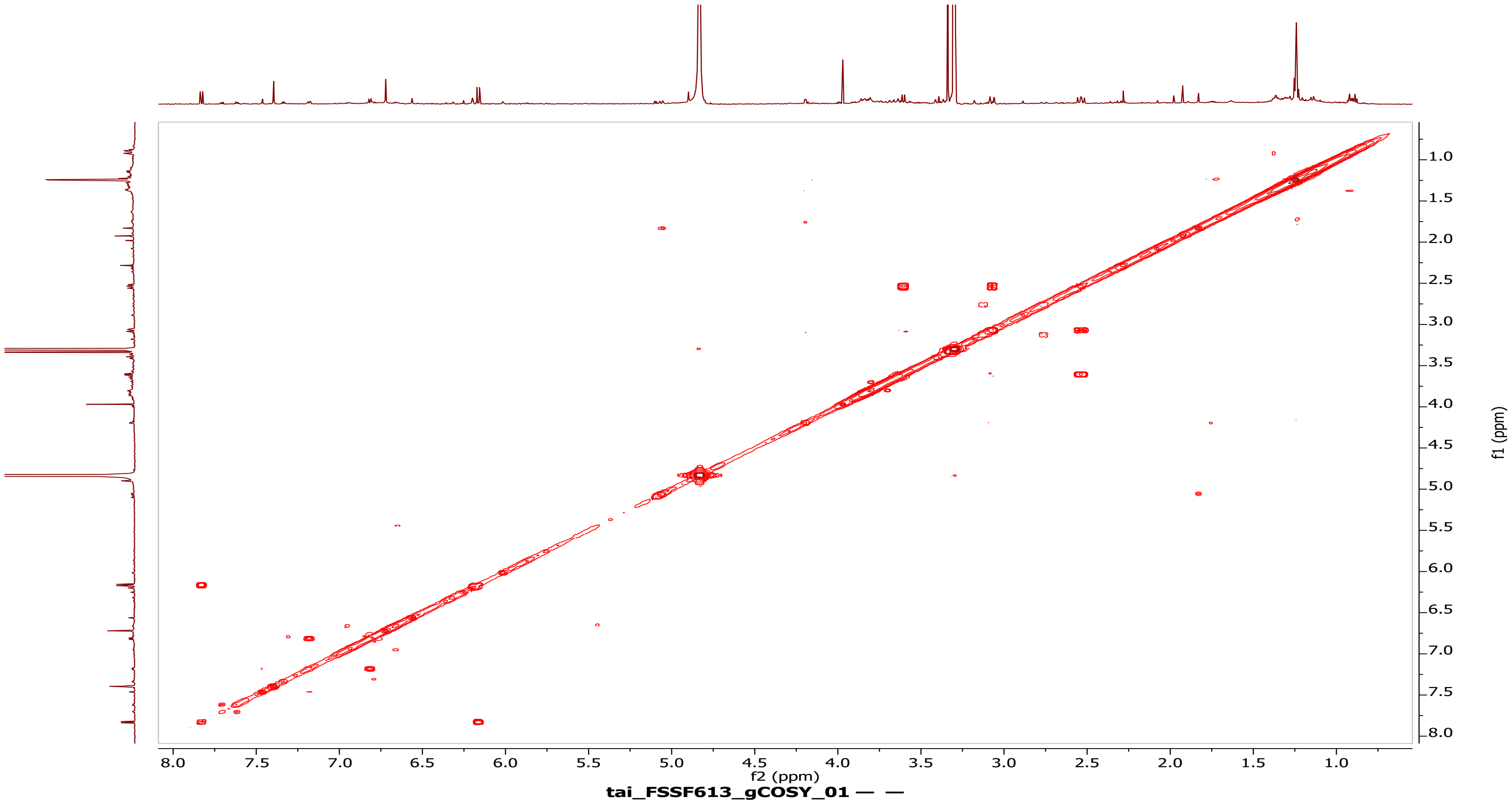

Figure S-29. COSY spectrum of compound **6**

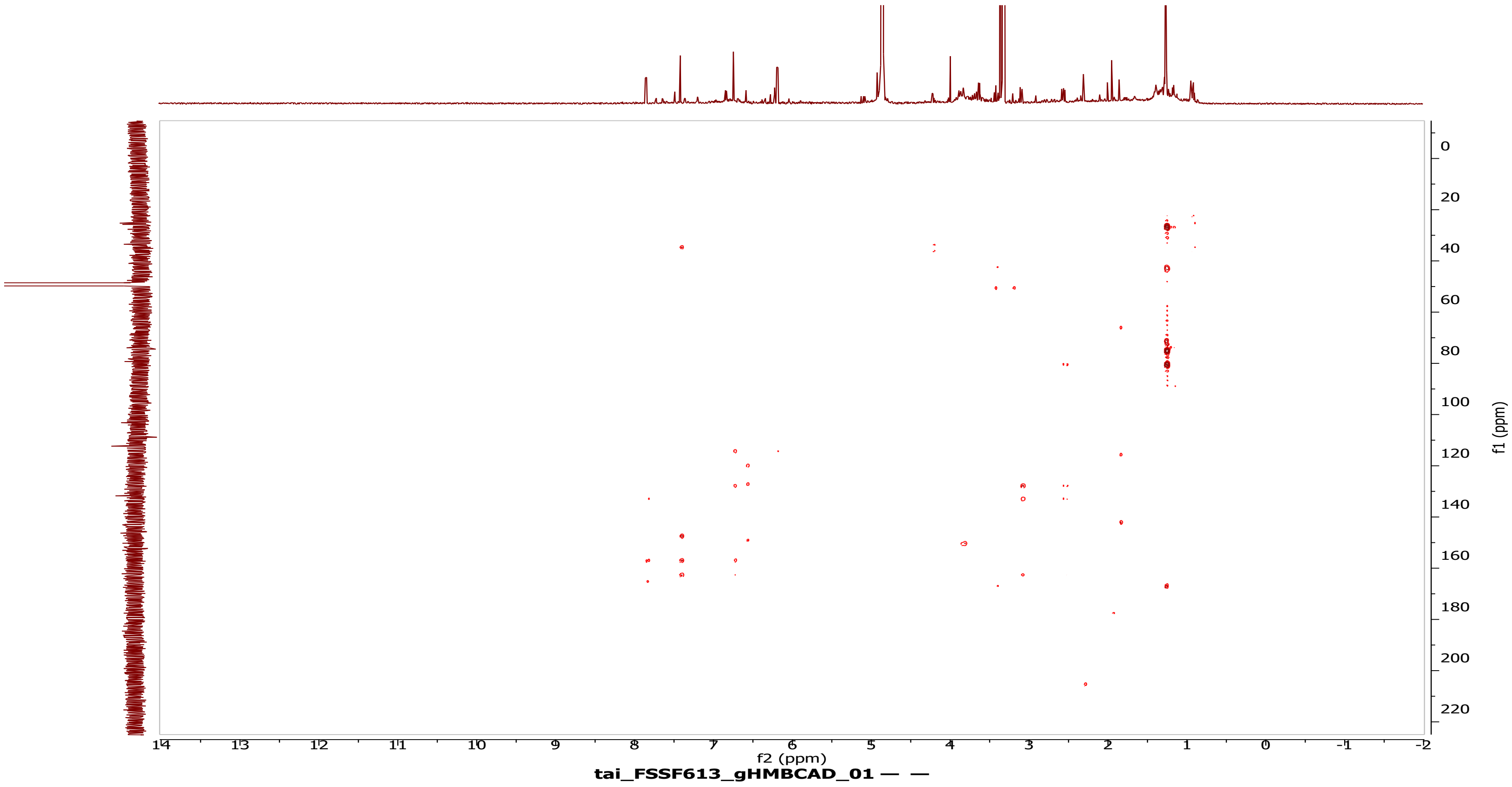

Figure S-30. HMBC spectrum of compound **6**

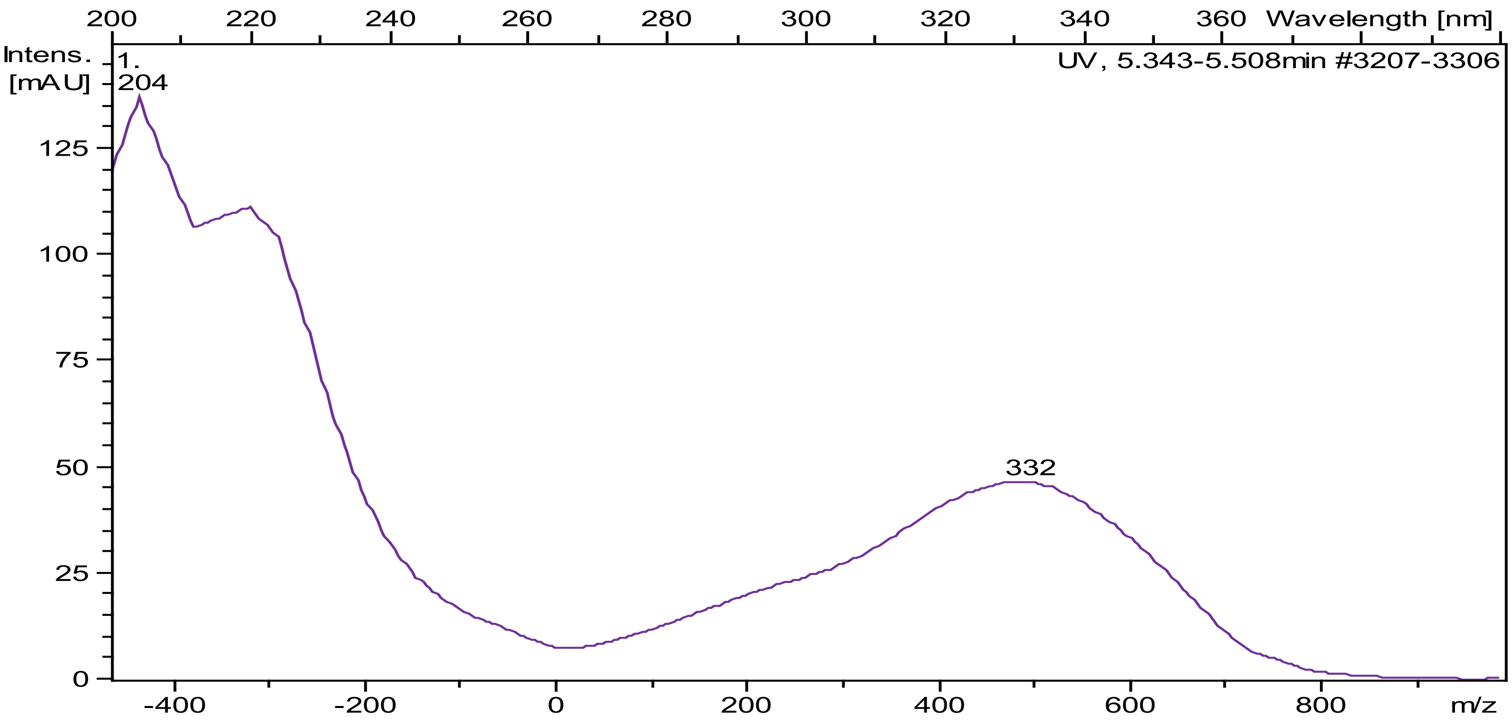

Figure S-31. LC-UV spectrum of compound **6**

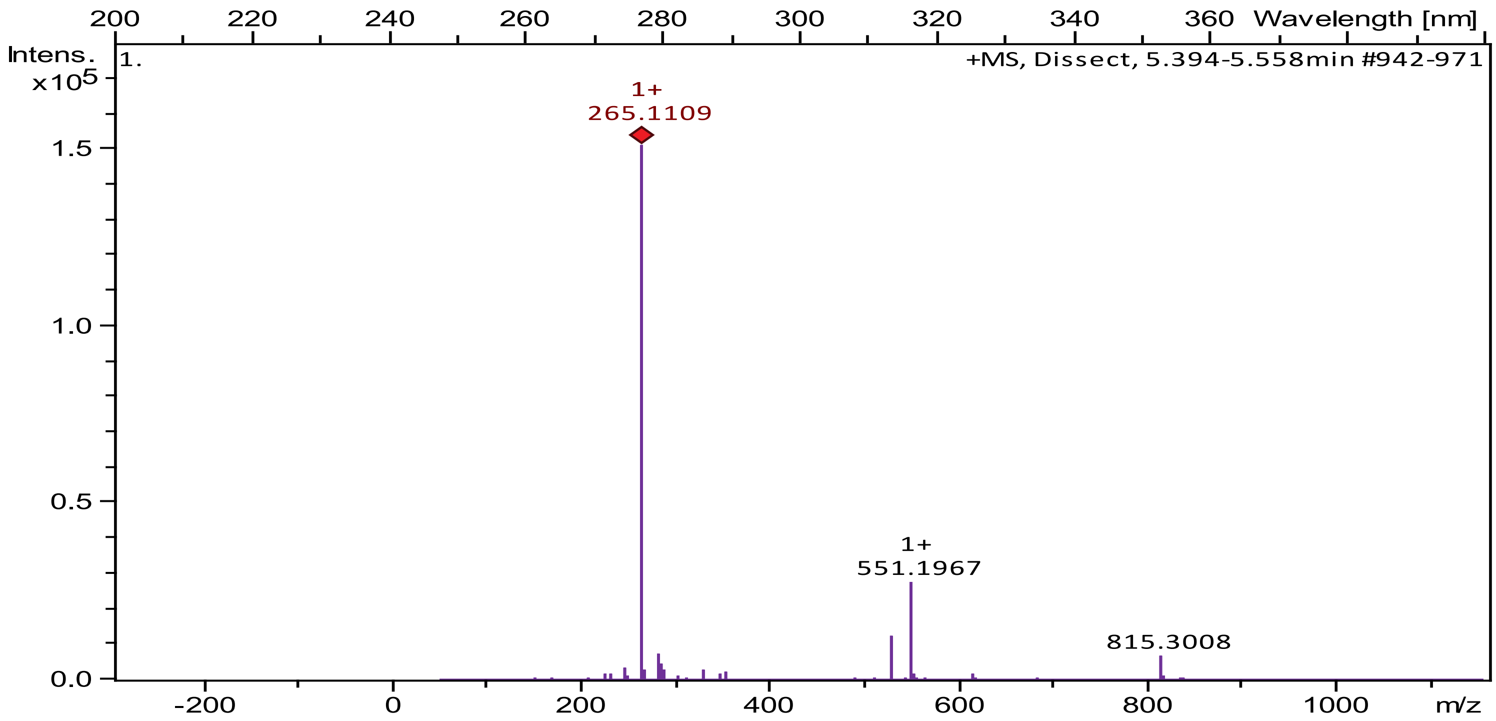

Figure S-32. HR-ESIMS spectrum of compound **6**

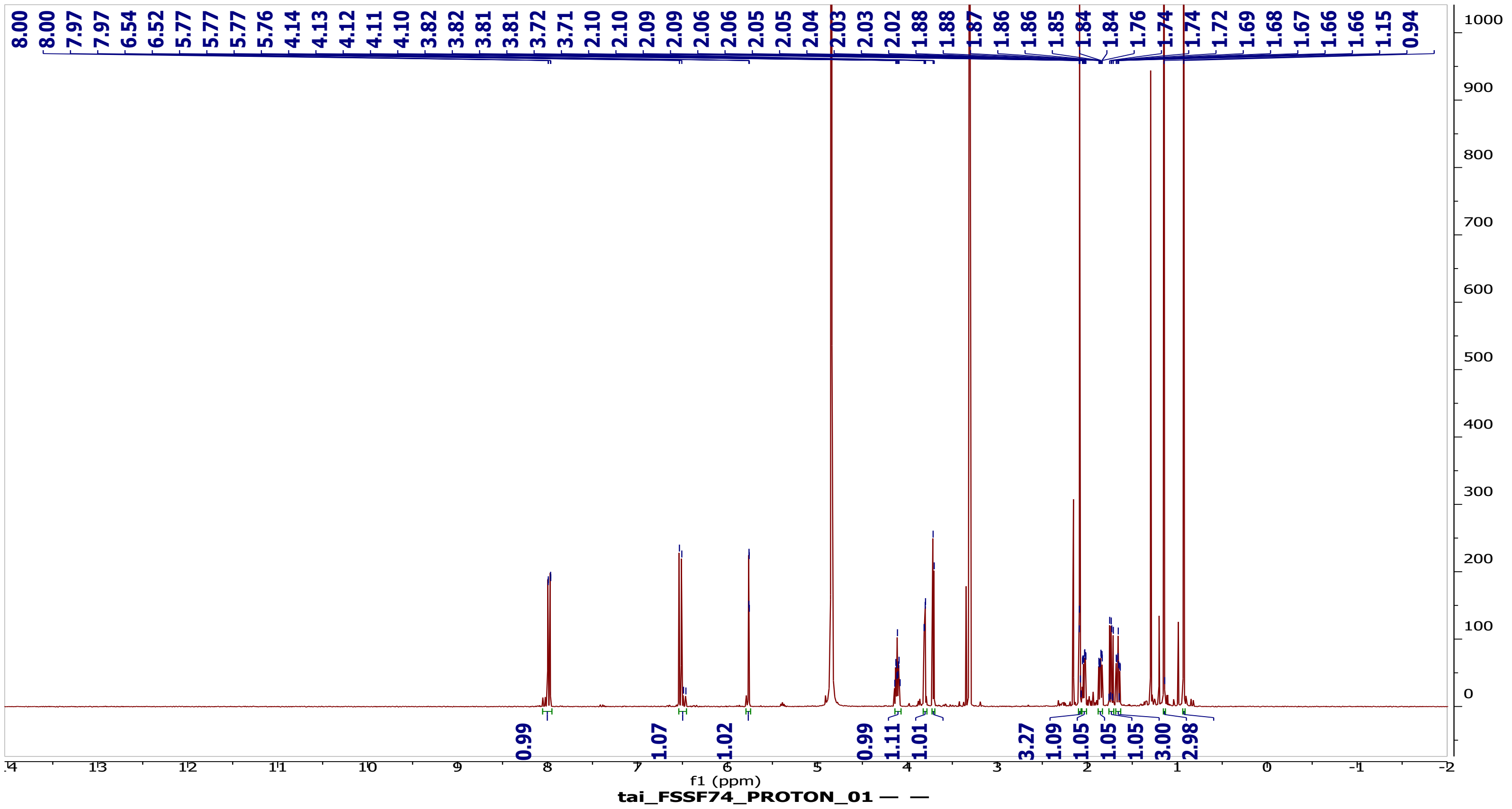

Figure S-33. ^{1}H-NMR spectrum for compound **7** (600 MHz, CD_3OD)

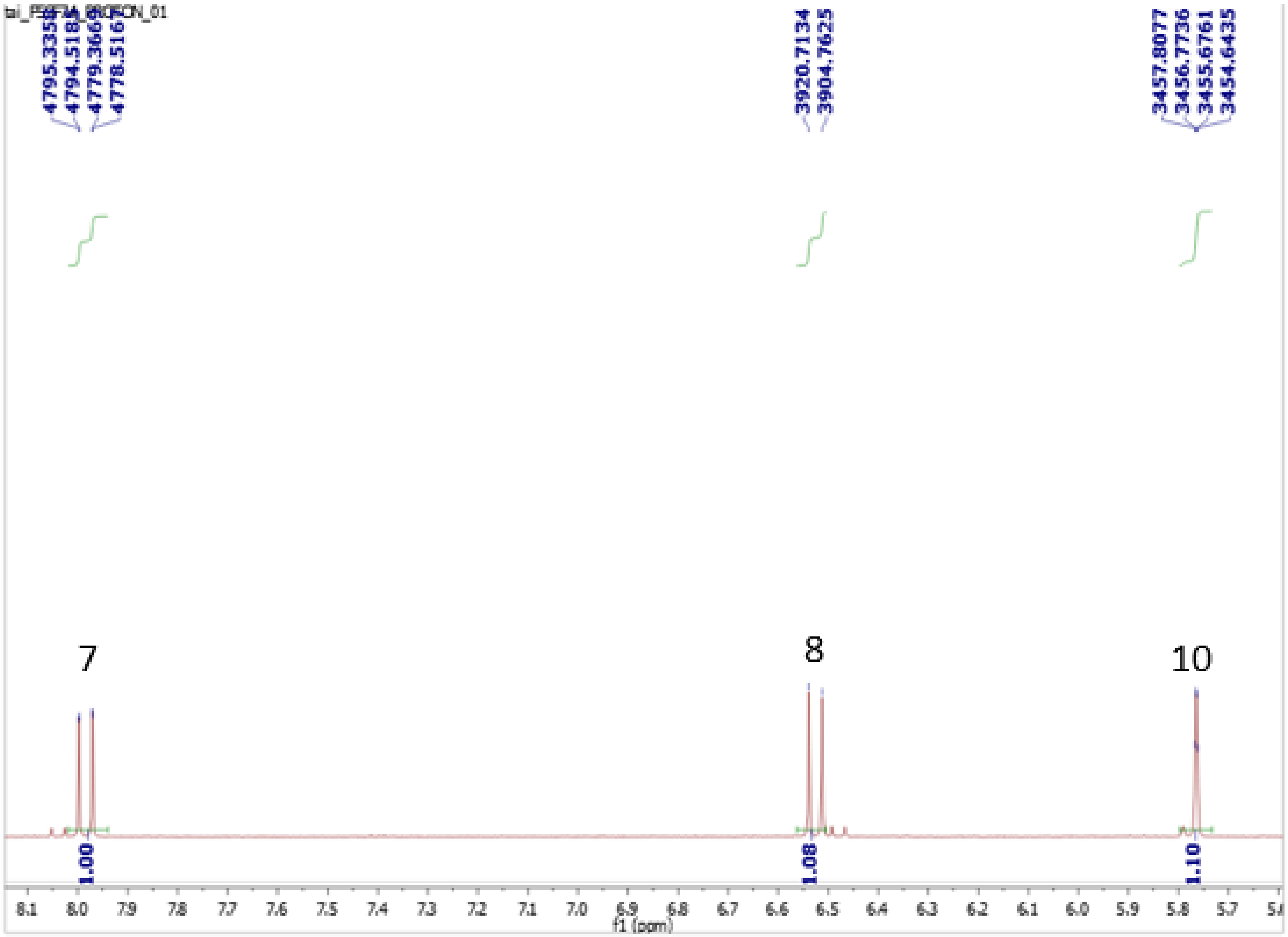

Figure S-34. ^{1}H-NMR spectrum of compound **7** (600 MHz, CD_3OD), expanded region showing the signals of H-7, H-8, H-10.

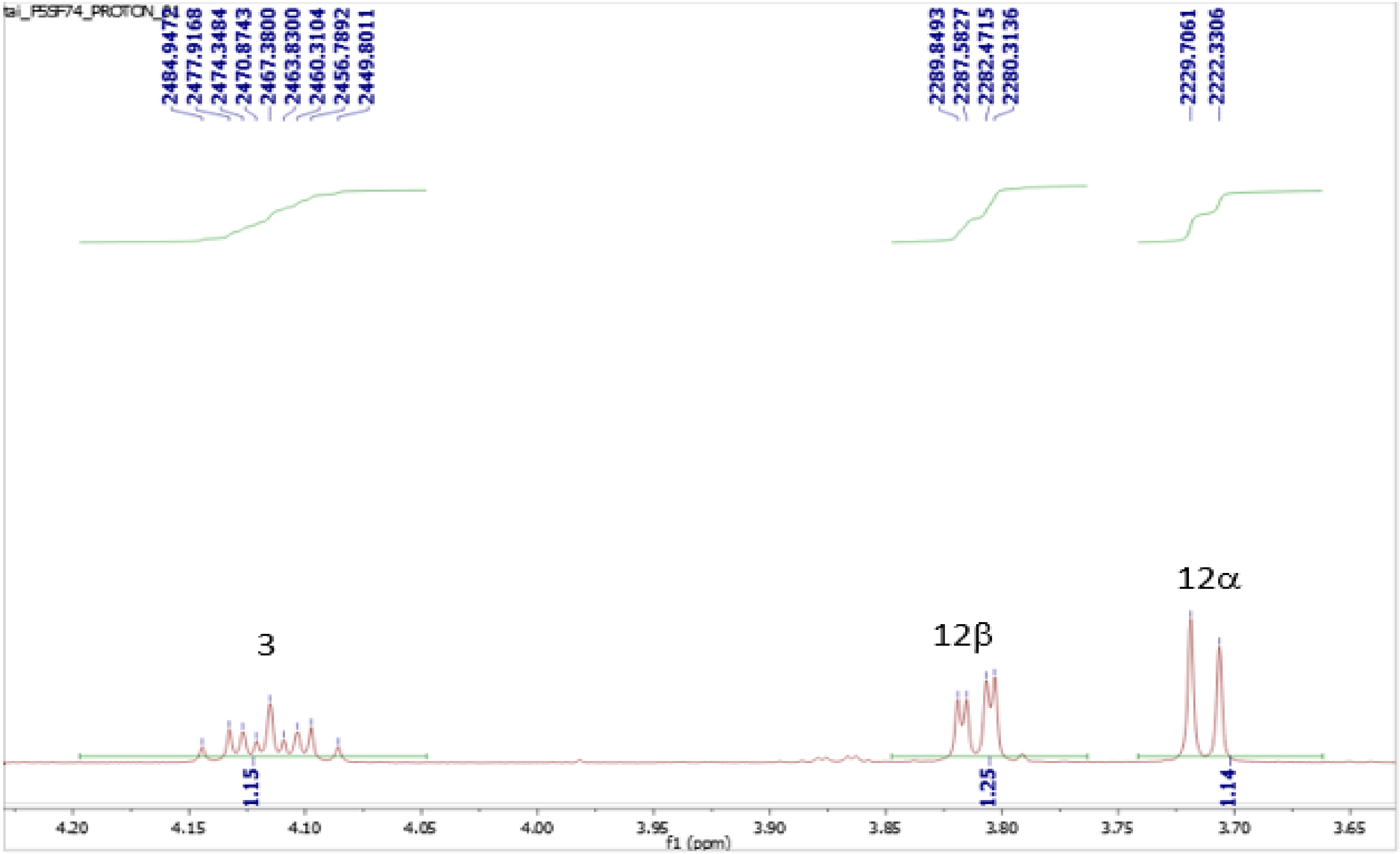

Figure S-35. ^{1}H-NMR spectrum of compound **7** (600 MHz, CD_3OD), expanded region showing the signals of H-3, H-12β, H-12α.

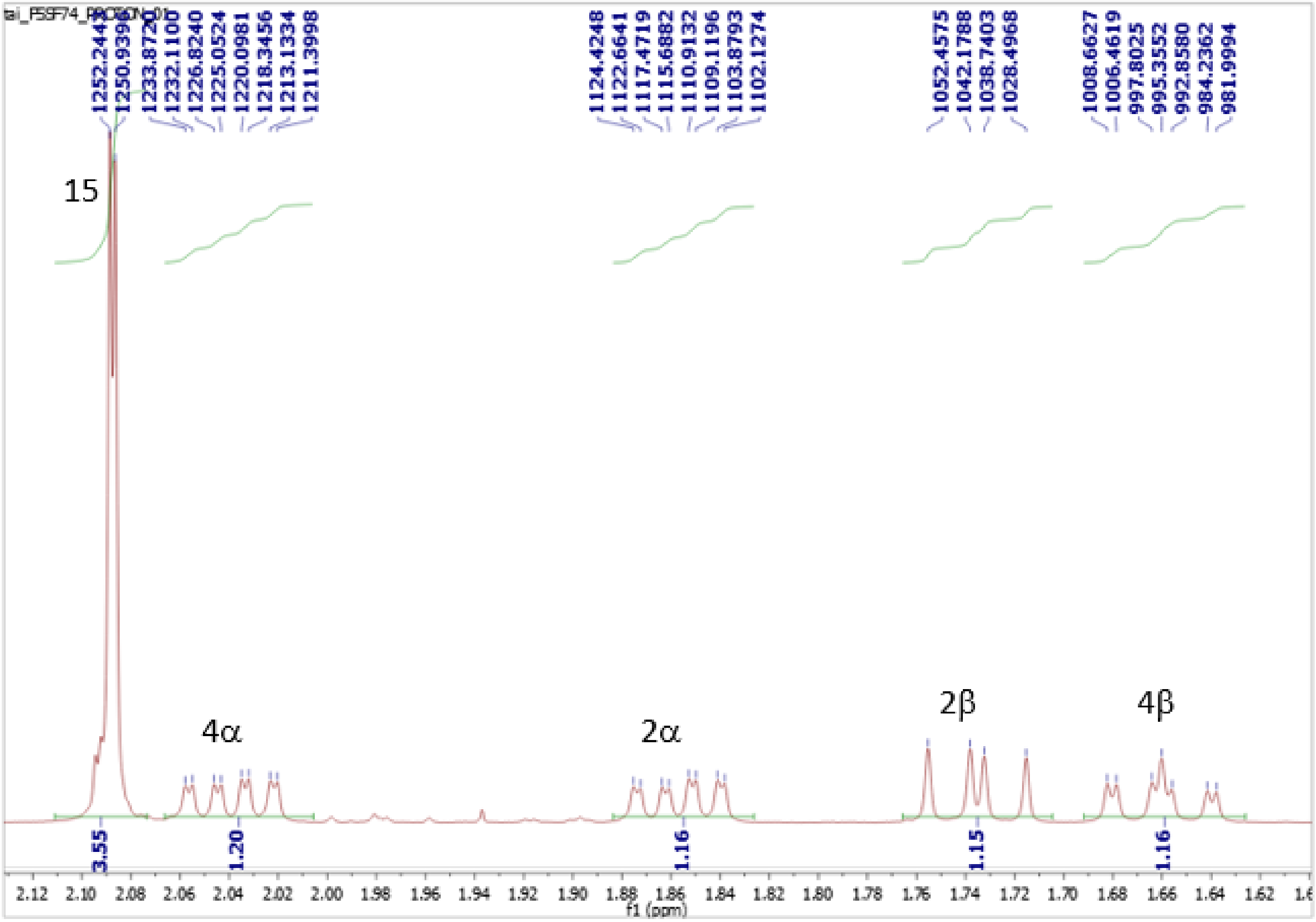

Figure S-36. ^{1}H-NMR spectrum of compound **7** (600 MHz, CD_3OD), expanded region showing the signals of CH_3-15, H-2α, H-4α, H-2β and H-4β.

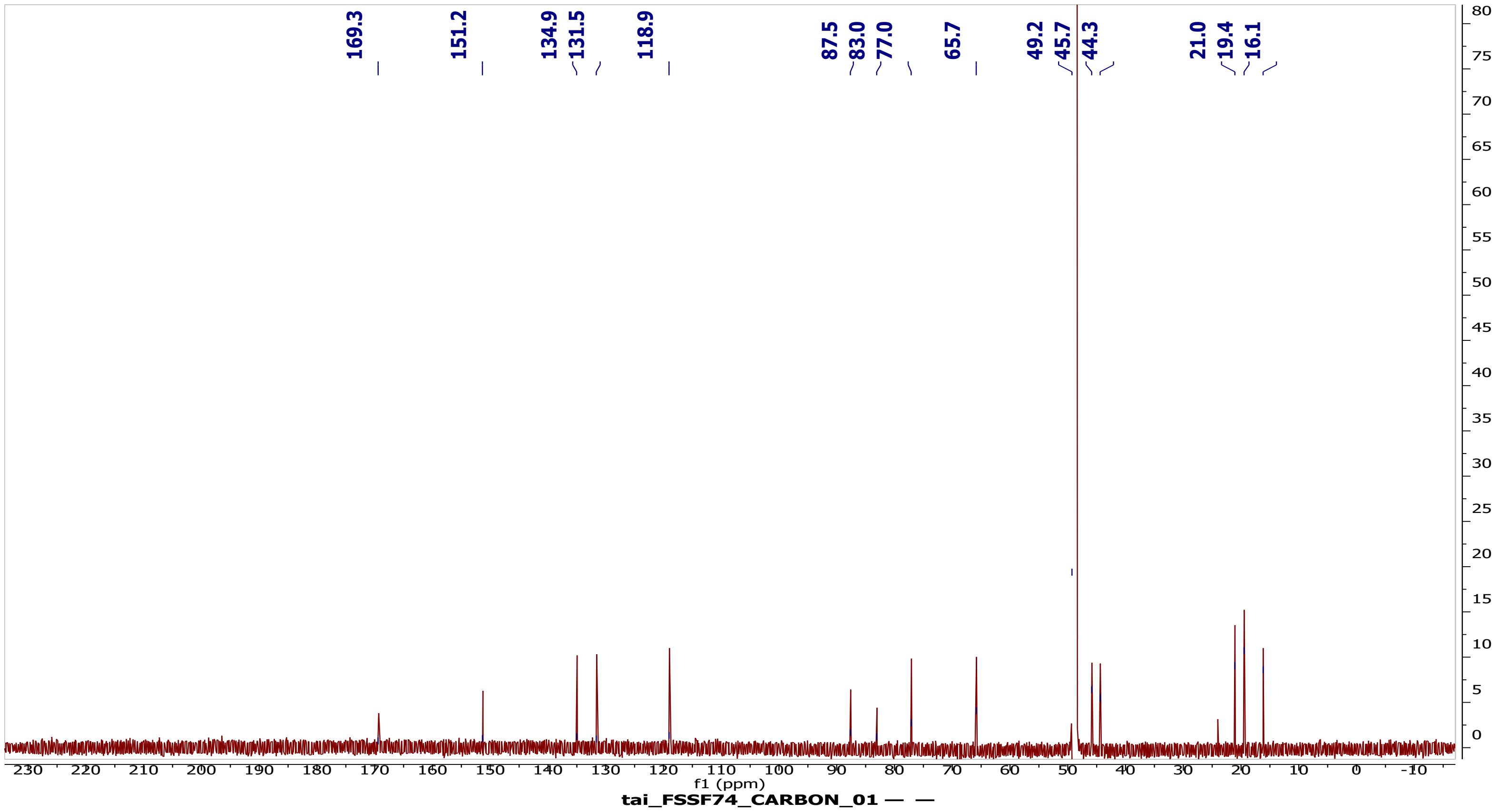

Figure S-37. ^{13}C-NMR spectrum of compound **7** (150 MHz, CD_3OD)

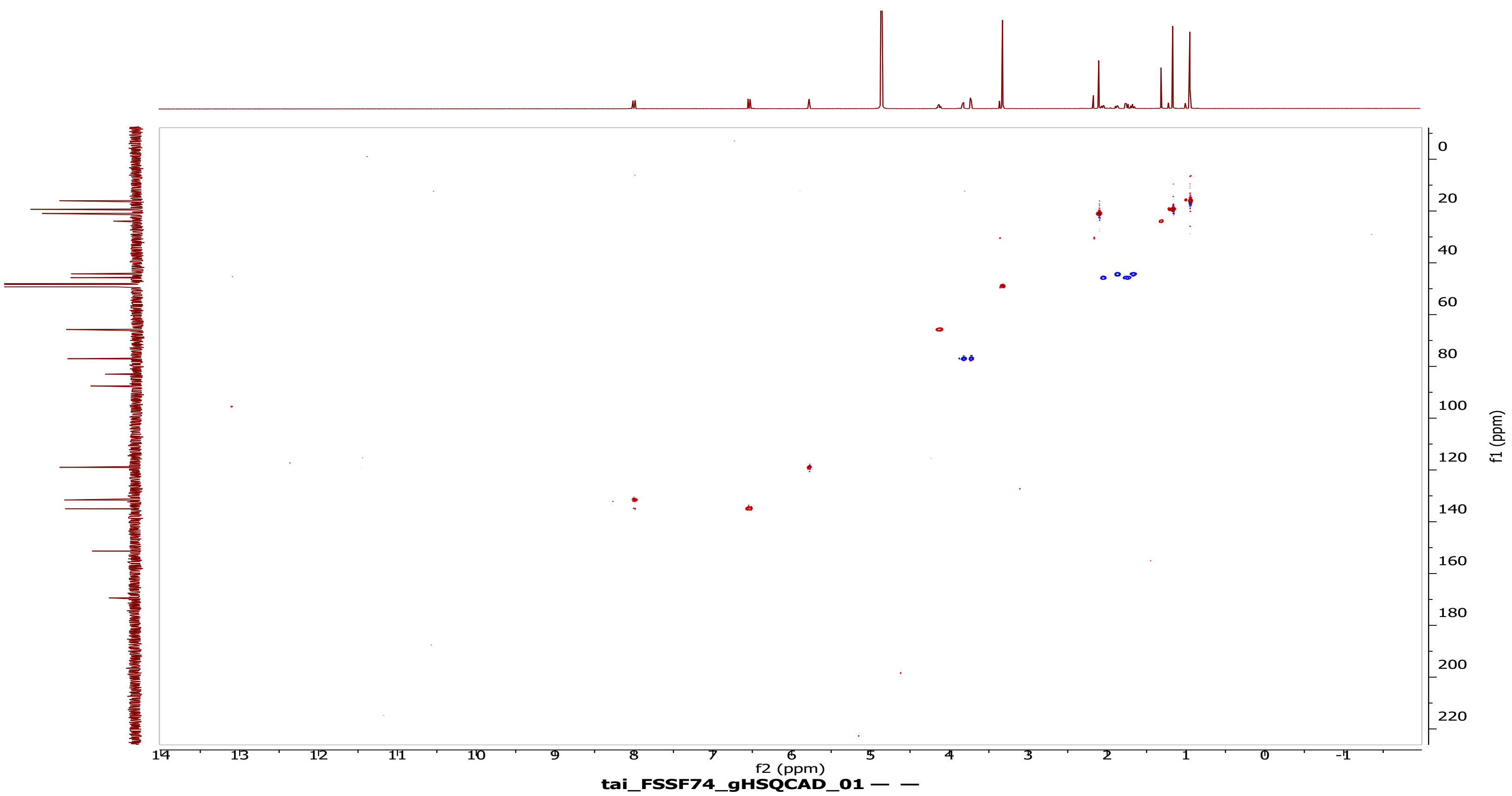

Figure S-38. HSQC spectrum of compound **7**

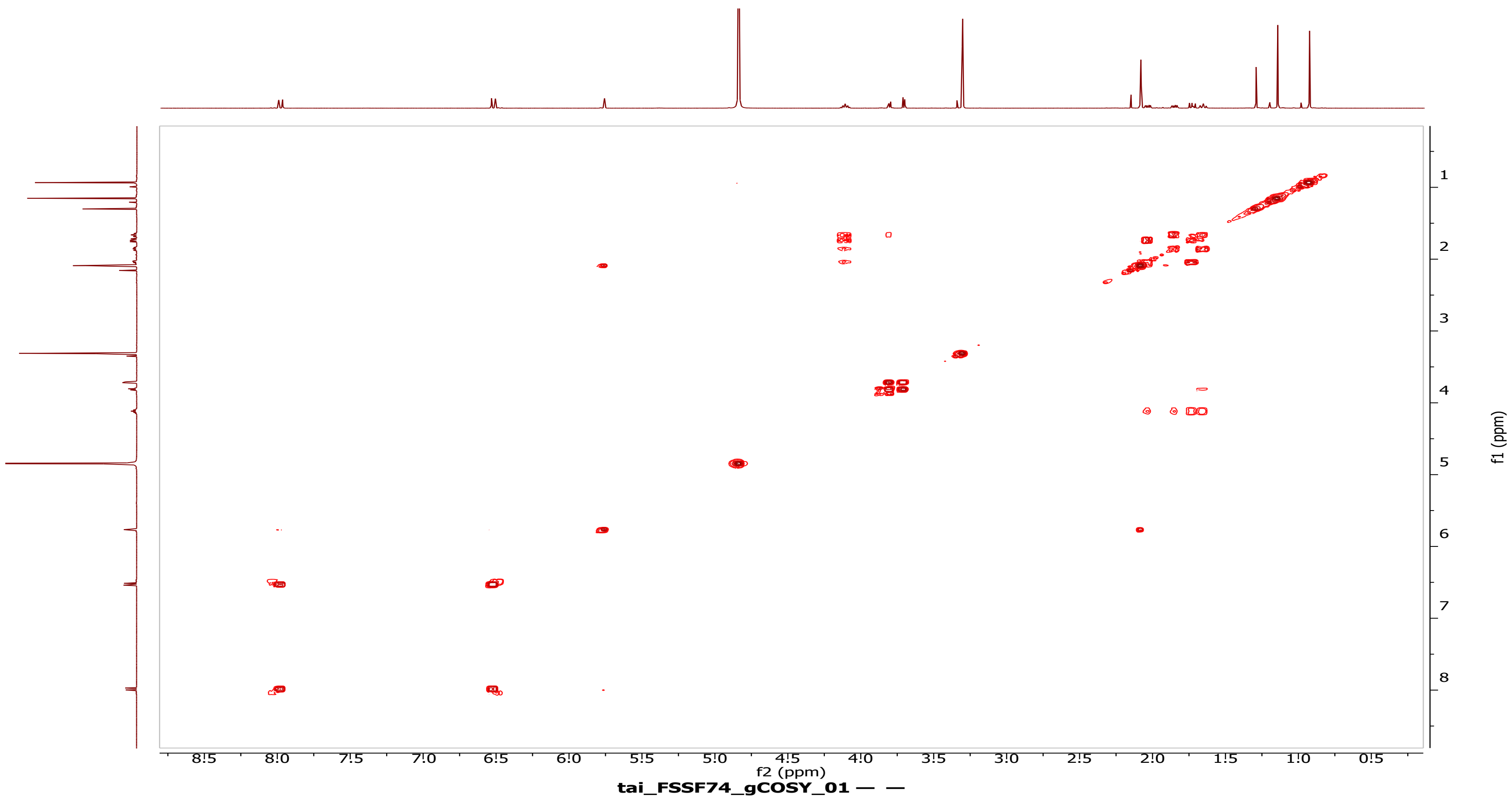

Figure S-39. COSY spectrum for compound **7**

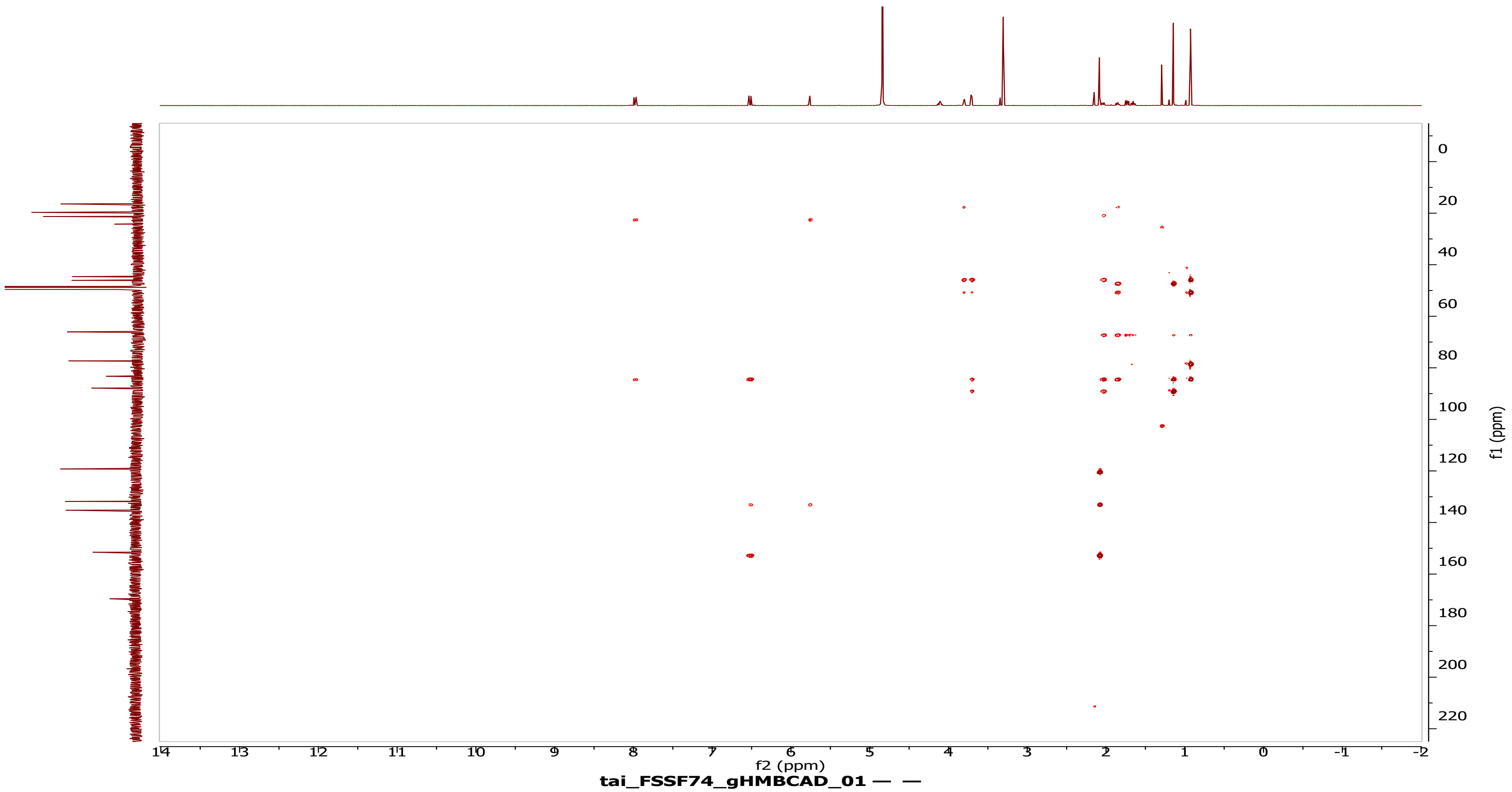

Figure S-40. HMBC spectrum of compound **7**

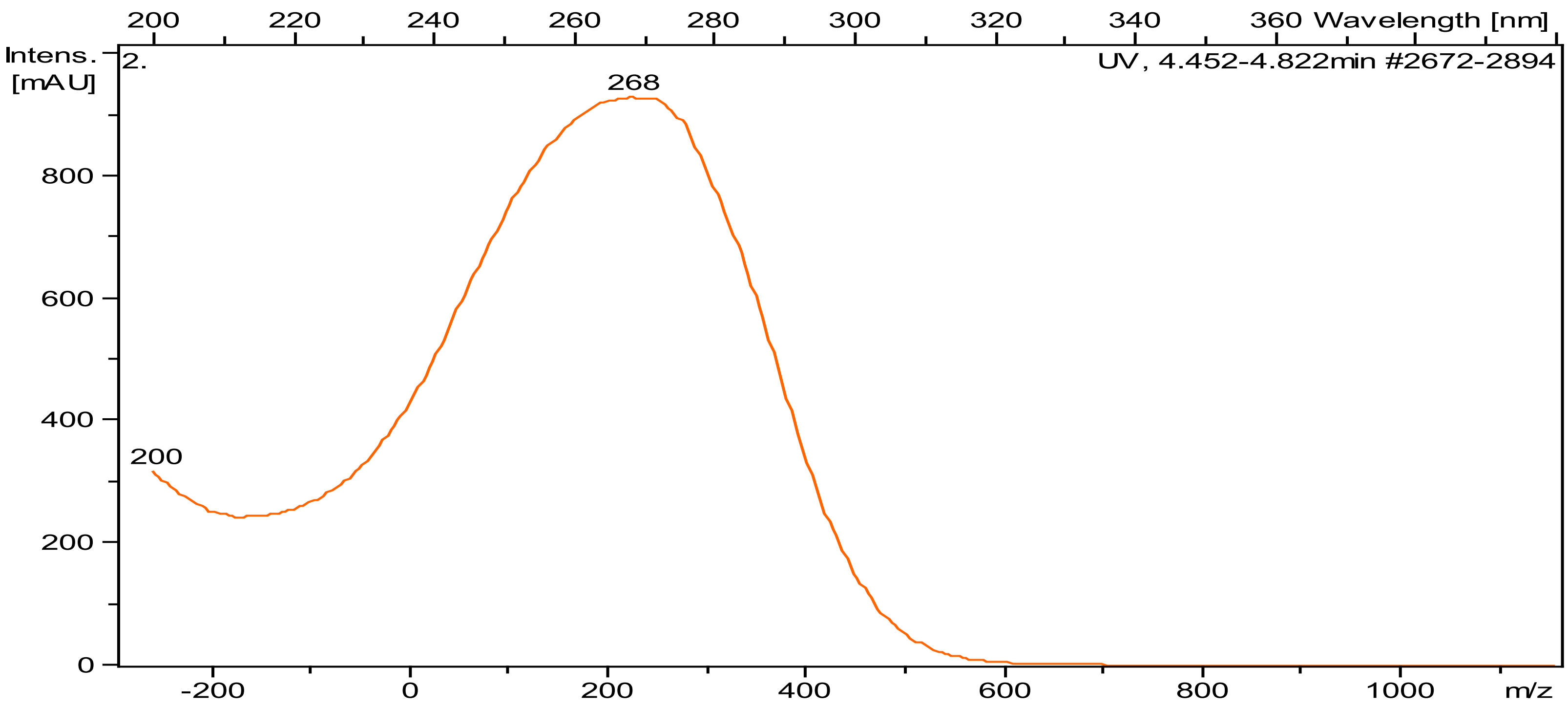

Figure S-41. LC-UV spectrum of compound **7**

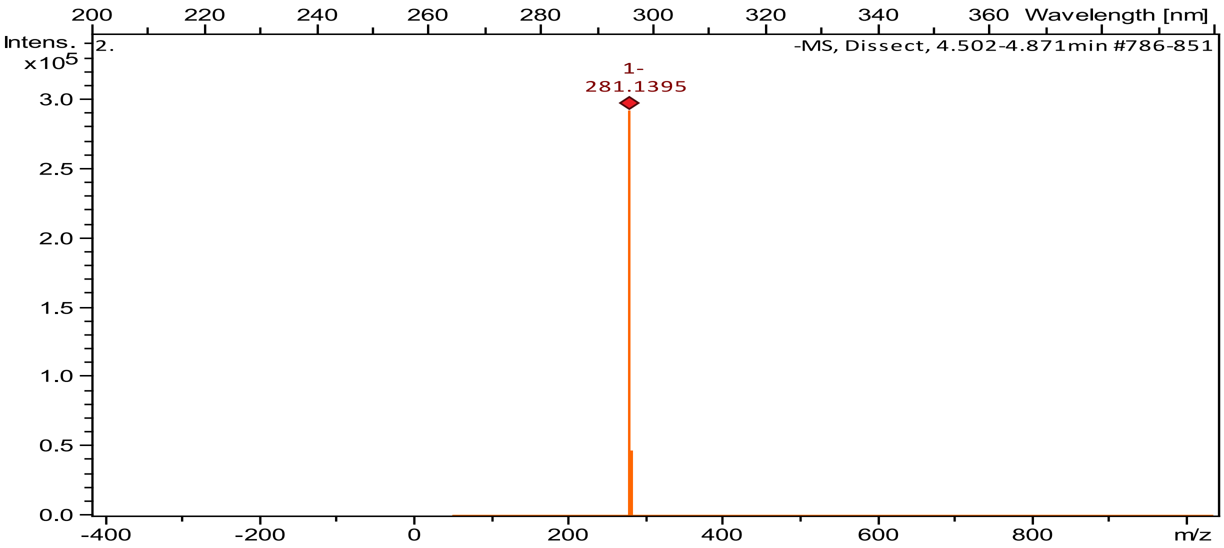

Figure S-42. HR-ESIMS spectrum of compound **7**
